# Assessment of four DNA fragments (COI, 16S rDNA, ITS2, 12S rDNA) for species identification of the Ixodida (Acari: Ixodida)

**DOI:** 10.1186/1756-3305-7-93

**Published:** 2014-03-03

**Authors:** Jizhou Lv, Shaoqiang Wu, Yongning Zhang, Yan Chen, Chunyan Feng, Xiangfen Yuan, Guangle Jia, Junhua Deng, Caixia Wang, Qin Wang, Lin Mei, Xiangmei Lin

**Affiliations:** 1 Institute of Animal Quarantine, Chinese Academy of Inspection and Quarantine, Beijing 100029, People’s Republic of China; 2 Institute of Plant Quarantine, Chinese Academy of Inspection and Quarantine, Beijing 100029, People’s Republic of China

**Keywords:** DNA Barcode, COI, Species identification, Ticks, 16S rDNA, ITS2, 12S rDNA, Nearest neighbour, BLASTn

## Abstract

**Background:**

The 5’ region of cytochrome oxidase I (COI) is the standard marker for DNA barcoding. However, COI has proved to be of limited use in identifying some species, and for some taxa, the coding sequence is not efficiently amplified by PCR. These deficiencies lead to uncertainty as to whether COI is the most suitable barcoding fragment for species identification of ticks.

**Methods:**

In this study, we directly compared the relative effectiveness of COI, 16S ribosomal DNA (rDNA), nuclear ribosomal internal transcribed spacer 2 (ITS2) and 12S rDNA for tick species identification. A total of 307 sequences from 84 specimens representing eight tick species were acquired by PCR. Besides the 1,834 published sequences of 189 tick species from GenBank and the Barcode of Life Database, 430 unpublished sequences representing 59 tick species were also successfully screened by Bayesian analyses. Thereafter, the performance of the four DNA markers to identify tick species was evaluated by identification success rates given by these markers using nearest neighbour (NN), BLASTn, liberal tree-based or liberal tree-based (+threshold) methods.

**Results:**

Genetic divergence analyses showed that the intra-specific divergence of each marker was much lower than the inter-specific divergence. Our results indicated that the rates of correct sequence identification for all four markers (COI, 16S rDNA, ITS2, 12S rDNA) were very high (> 96%) when using the NN methodology. We also found that COI was not significantly better than the other markers in terms of its rate of correct sequence identification. Overall, BLASTn and NN methods produced higher rates of correct species identification than that produced by the liberal tree-based methods (+threshold or otherwise).

**Conclusions:**

As the standard DNA barcode, COI should be the first choice for tick species identification, while 16S rDNA, ITS2, and 12S rDNA could be used when COI does not produce reliable results. Besides, NN and BLASTn are efficient methods for species identification of ticks.

## Background

Accurate identification of species of ticks is important to control tick-borne diseases and has traditionally been achieved through morphological criteria of ticks in adult stage [1-6]. However, species identification by morphological data can be difficult especially when the specimens are physically damaged, engorged with blood, or in subadult stages (i.e., eggs, larvae, or nymphs) [7,8].

The 5’ region of the mitochondrial DNA gene cytochrome C oxidase subunit I (COI) is the standard marker for DNA barcoding [9-11]. Our previous study has demonstrated that COI and 16S ribosomal DNA (rDNA) were reliable in species identification of ticks and a DNA barcoding system for ticks based on three DNA markers (COI, 16S rDNA, and 18S rDNA) was developed [12]. However, there are still several problems with this DNA barcoding system. First, the primer pair COI-F/COI-R is not efficient in the recovery of COI from tick specimens; second, our previous study only focused on the tree-based method [12] and did not evaluate the efficiency of BLASTn and distance methods, which have proved to give reliable species identification rates; finally, compared with the DNA barcoding method based on a single molecular marker, the barcoding system based on three DNA markers was time consuming and noneconomic. Besides, several researches have proved that some DNA fragments were superior to COI in species identification of certain taxa and the amplification of COI from some species often failed [13-15]. Until now, whether COI is the most suitable DNA marker to discriminate species of ticks is still unknown. At the same time, 12S rDNA and ITS2 have significantly advanced our understanding of the evolution of ticks [3,16-22]. However, these DNA markers have not been tested for species identification of ticks.

To resolve the above problems, we evaluated the performance of four DNA fragments (COI, 16S rDNA, ITS2 and 12S rDNA) for species identification of ticks. In the present study, several sets of COI primers for ticks with published sequences including COI-F/COI-R were tested for their effectiveness in amplifying COI sequences from 84 specimens representing four tick genera. 307 sequences (including COI, 16S rDNA, ITS2 and 12S rDNA) were amplified and sequenced. Besides the 307 sequences, the final data set also consists of 1,834 published sequences and 430 unpublished sequences screened by Bayesian analyses. Genetic divergence of each candidate DNA fragment was determined using six parameters (average inter-specific distance, theta prime, the smallest inter-specific distance, average intra-specific distance, theta, and coalescent depth). Moreover, efficiencies of the four DNA fragments for tick species identification were evaluated following four methods: the NN (Nearest neighbor), BLASTn, liberal tree-based and liberal tree-based (+threshold).

## Methods

### Taxon sampling

**Figure 1:**
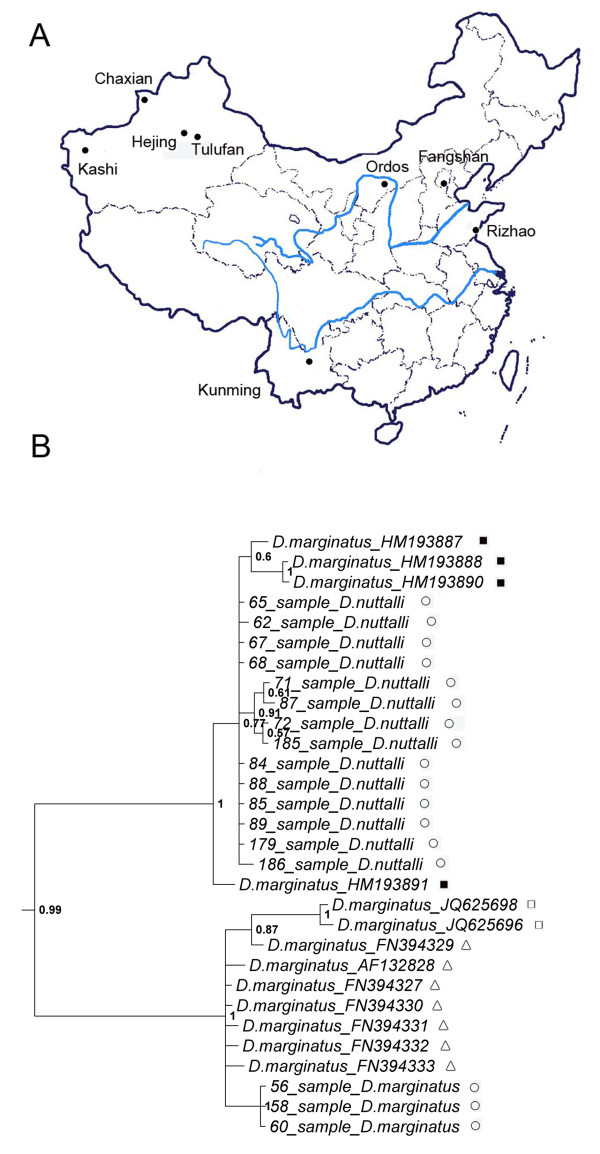
**Data collection and phylogenetic screening of sequences. (A)** Localities where the 84 tick specimens used in this study were collected. **(B)** Phylogeny of ticks resulting from Bayesian analysis of COI. Numbers on branches are posterior probabilities. ○, sequence amplified from our specimens; △, previously published sequences; □, unpublished sequences included in data set; ■, misplaced unpublished sequences excluded from data set.

The 84 tick specimens used in this study were collected from various field sites of China (Figure 1A). Ticks from Yunnan Province were taken from cattle on a farm in the suburb of Kunming. Ticks from Xinjiang Autonomous Region were collected from sheep on farms of Kashi city, Hejing county, Tulufan city, and Chaxian county. Ticks from Inner Mongolia Autonomous Region were collected from sheep on farms in the Ordos grassland. Ticks from Beijing were collected by sweep netting the vegetation on a farm in the suburb of Fangshan District. Ticks from Shandong Province were collected from cattle and goats on a farm in the suburb of Rizhao city.

The species of adult tick specimens were identified by the specialist through the morphological and molecular data [23]. The specimens in the subadult stages were obtained from mated ticks in adult stage and the species of these juveniles were determined through identification of their “parents”. The detailed information of specimens (specimen codes, species, collecting locations, development stages) is shown in Additional file 1: Table S1. All specimens were preserved in 100% ethanol.

### DNA extraction and PCR

Ethanol-preserved ticks were rinsed in distilled water, and total DNA was extracted using a DNeasy blood and tissue kit (Qiagen GmbH, Hilden, Germany) following the manufacturer’s protocol. The fragments of COI, 16S rDNA, ITS2 and 12S rDNA were amplified by PCR using the primers listed in Additional file 2: Table S2.

Each 50 μl PCR solution contained 25 μl of 2 × PCR Buffer for KOD FX Neo (1.75 mM final concentration of MgCl_2_), 10 μl of 2 mM dNTPs, 3 μl of primer mix (0.3 μM final concentration of each primer), 1 μl KOD FX Neo polymerase (1 unit), 2 μl DNA template (about 200 ng genomic DNA), and distilled water. PCR assays were conducted using a GeneAmp PCR System 9700 thermal cycler (Applied Biosystems, Foster City, CA, USA). The DNA size marker DL2000 (Takara, Dalian, China) was used to estimate the length of the PCR amplicons.

COI was amplified with COI-F/COI-R using a touchdown PCR protocol, as follows: initial denaturation (94°C, 5 min); followed by five cycles of 94°C for 30 s, 52°C for 30 s, and 68°C for 1 min; five cycles of 94°C for 30 s, 50°C for 30 s, and 68°C for 1 min; five cycles of 94°C for 30 s, 48°C for 30 s, and 68°C for 1 min; 25 cycles of 94°C for 30 s, 46°C for 30 s, and 68°C for 1 min; followed by a final extension step of 68°C for 5 min. The amplification of COI gene using published primer pairs (Additional file 2: Table S2) was carried out as previously described [21,22,24,25]. The protocol for the 16S rDNA gene amplification using 16S-F/16S-R1 was: initial denaturation (94°C, 5 min); followed by five cycles of 94°C for 30 s, 49°C for 30 s, and 68°C for 30 s; five cycles of 94°C for 30 s, 47°C for 30 s, and 68°C for 30 s; five cycles of 94°C for 30 s, 45°C for 30 s, and 68°C for 30 s; 25 cycles of 94°C for 30 s, 43°C for 30 s, and 68°C for 30 s; followed by a final extension step of 68°C for 5 min. The protocol for ITS2 amplification using ITS2-F/ITS2-R was: initial denaturation (94°C, 5 min); followed by thirty-five cycles of 94°C for 30 s, 55°C for 30 s, and 68°C for 2 min; followed by a final extension step of 68°C for 5 min. The amplification of 12S rDNA by T1B/T2A (Additional file 2: Table S2) followed the protocol described in a previous study [26].

### Data acquisition and sequence alignment

For the field-collected specimens, DNA was amplified and sequences were obtained for the COI (675 bp or 820 bp), 16S rDNA (454 bp), ITS2 (1200 to 1600 bp) and 12S rDNA (250 to 360 bp). The PCR amplicons were sequenced at BGI Tech Inc. (Beijing, China).

As to COI, the GenBank accession numbers are JX051119 to JX051164, KF583568 to KF583579, and KC203341 to KC203362. As to 16S rDNA, the accession numbers are KC203338 to KC203362, JX051062 to JX051118. All ITS2 sequences of specimens were deposited in GenBank under accession numbers KC203363 to KC203433. As to 12S rDNA, the accession numbers are KF583582 to KF583655. Amplification success rate was measured based on the proportion of samples that produced sequences of the appropriate length. These sequences were further evaluated for their utility in species identification.

Sequences of COI, 16S rDNA, ITS2 and 12S rDNA of ticks were downloaded from BOLD (http://www.barcodinglife.org/) and GenBank (http://www.ncbi.nlm.nih.gov/genbank/). Some sequences were methodologically omitted in this study: the COI sequences that were shorter than 450 bp; the 16S rDNA sequences that were shorter than 350 bp; the ITS2 sequences that cover less than 70% of full length; the 12S rDNA sequences that were shorter than 240 bp.

DNA sequences were assembled and edited in MEGA 5.0 [27]. Sequences of the same gene were aligned using ClustalW [28] in MEGA 5.0 with default parameters (open gap penalty = 10.0, extend gap penalty = 5.0) and the resulting matrix was then manually corrected. COI, 16S rDNA, ITS2 or 12S rDNA sequences constituted different datasets for each genus of ticks. Analyses were conducted with these datasets. All the sequences of the same gene region were trimmed to the same length to allow comparisons within each dataset.

Sequences were assigned quality scores using PHRED as implemented in Codon Code Aligner 3.5.6 (Codon Code Corp.). Consensus sequences were generated, and for each DNA region and sample, we recorded the length of the consensus sequence, the percentage of the consensus sequence length for which a bidirectional read was available and the percentage of bases in the consensus sequence with qv >20 (Additional file 3: Appendix S1, Supporting information).

### Genetic divergence analysis through six parameters

Genetic distances were calculated using the Kimura two parameters (K2P) distance method [29] as implemented in MEGA 5.0.

Three parameters were used to characterize inter-specific divergence [30,31]: (1) average inter-specific distance (K2P distance) between all species in each genus with at least two species; (2) average theta prime, where theta prime is the mean pairwise distance within each genus with more than one species, thus eliminating biases associated with different numbers of species among genera; and (3) smallest inter-specific distance, i.e. the minimum inter-specific distance within each genus with at least two species.

In addition, three parameters were used to determine intra-specific variation: (1) average intra-specific difference (K2P) between all samples collected within each species with more than one individual; (2) theta, where theta is the mean pairwise distance within each species with at least two representatives; theta eliminates biases associated with unequal sampling among species; (3) average coalescent depth, which is the maximum intra-specific distance within each species with at least two individuals.

### Bayesian analyses

For COI, Bayesian analyses were performed using MrBayes 3.2 [32], with the data partitioned into three sets by codon position. Models for each partition were selected using MrModeltest 2.2 [33]; these were determined to be GTR + I + G for codon positions 1, GTR + I for codon position 2, and TrN + G for codon position 3. For 16S rDNA and ITS2, the model was determined to be GTR + I + G. For each Bayesian analysis, two runs were performed simultaneously, each with four Markov chains (one cold, three heated), which ran for 1,000,000 generations. The first 250,000 generations were discarded from analysis (burnin) and every 1000th tree was sampled to calculate 50% majority-rule consensus trees with posterior probabilities for nodes.

### Data sets utilized in this study

The data set 1 includes 307 sequences generated from the 84 tick specimens, 1834 published sequences from BOLD and GenBank and 430 unpublished sequences from GenBank. Overall, data set 1 consists of 873 16S rDNA sequences from 133 species, 465 COI sequences from 69 species, 427 ITS2 sequences from 70 species and 806 12S rDNA sequences from 99 species (Additional file 4: Table S3).

The DNA sequences were also grouped into two other data sets: set 2 consists of the species with multiple accessions (irrespective of congeneric species); set 3 includes 256 sequences obtained from the 68 adult specimens and 1834 published sequences from BOLD and GenBank. All the data sets are available in Additional file 5: Appendix S2.

### Investigation of species boundaries

“False negatives” are specimens coming from two different species that are classified within the same species; “False positives” are specimens belonging to the same species that are classified in two different species. By varying the threshold value from 0% to 15%, the cumulative distribution functions of “False negatives” and of “False positives” were drawn. We used the number of these errors to suggest a ministration of their sum in order to obtain an optimal threshold value.

### Methods utilized for species identification

#### Nearest neighbor (NN)

Genetic distances were calculated using the K2P distance method [29] as implemented in MEGA 5.0. In the analysis, the query sequence was assigned to the species of the sequence in the reference database, which has the smallest genetic distance from the query sequence. If nearest neighbours were from more than one species the query’s identification was considered uncertain.

#### BLASTn

Identification based on BLASTn was performed using NCBI software version 2.2.28+ [34]. Up to 100 hits with at least 80% identity were returned for each query sequence, which was assigned to the species of the sequence associated with its best hit (highest bit score). If more than one species was associated, the query’s identification was considered uncertain.

#### Liberal tree-base method

When the query sequence (Q) was either sister to ((X, X), Q), or within ((X, Q), X) a monospecific clade, the ID is that of the species in the clade (X); otherwise it was considered uncertain.

#### Liberal tree-based method (+threshold)

As liberal tree-based method but requiring that the nearest reference sequence is less than a distance threshold; otherwise, it was considered uncertain.

### Sequence identification success

We evaluated the performance of four DNA markers in terms of their identification success rates with data sets of ticks in this study. Identification success rate was defined as “Sequence identification success rate”, scored as the number of correctly identified query sequences divided by total number of sequences for each dataset.

### Statistical tests

We evaluated the influence of i) method, and ii) DNA fragment on sequence identification success rates. The significances were determined by the Duncan’s Multiple Range test using the SAS software version 9.2 (SAS Institute Inc, Cary, North Carolina, USA).

## Results

### Primer performance and sequencing quality

Our previous study showed that the redesigned primer, COI-F/COI-R, was not efficient in amplifying COI from tick specimens [12]. In this study, several sets of COI primers for ticks with published sequences including COI-F/COI-R were evaluated for their effectiveness in amplifying COI sequences from 84 specimens representing four tick genera. The primer pairs included HCO1490/LCO2198, HCO2064/HCO1215, Cox1F/Cox1R and TY-J1449/C1-N-2312. Cox1F/Cox1R gave the highest rate of COI sequence recovery (94.0%) and COI-F/COI-R gave the second highest rate (78.6%) (Additional file 6: Table S4). However, all of the other three sets of primers gave poor COI recovery rates ranging from 14.3% to 27.4%.

The amplification success rate for 16S-F/16S-R1 was 97.6% (i.e., 82 out of 84 specimens).

The ITS2 sequences of ticks were highly variable. Hence, new primers (ITS2-F/ITS2-R) for ITS2 were designed based on the conserved 5.8S rDNA and 28S rDNA regions adjacent to ITS2. The amplification success rate for ITS2-F/ITS2-R was 84.5% (71 out of the 84 tick specimens). For 12S rDNA, T1B/T2A primer pair successfully amplified 74 12S rDNA sequences from 84 specimens (88.1%).

To investigate whether particular DNA sequences of certain tick species are difficult to amplify, we carefully examined the DNA regions (COI, 16S rDNA, ITS2 and 12S rDNA) that failed to be amplified from the tick specimens used in this study. The detailed information is provided in Additional file 7: Table S5. Our results showed that at least one of the four DNA regions was successfully amplified from all of the 84 tick specimens (Additional file 1: Table S1). Nevertheless, 12S rDNA from *Dermacentor nuttalli*, ITS2 from *Hyalomma asiaticum* (*Hy. asiaticum*) and ITS2 from *Hy. anatolicum* were all relatively difficult to amplify.

The sequence quality of amplified genomic regions was highest for 16S rDNA, followed by COI, 12S rDNA, and ITS2. The read lengths and quality scores for each unidirectional and bidirectional sequence are given in Additional file 3: Appendix S1 (Supporting information).

### Screening COI, 16S rDNA, ITS2 and 12S rDNA sequences of ticks from BOLD and GenBank

Genetically closely related species were required to benchmark the resolution power of the four DNA markers at the species level. To resolve this issue, the tick sequences from GenBank and BOLD system were added to our data sets.

First, COI, 16S rDNA, ITS2 and 12S rDNA sequences of ticks available in GenBank and BOLD (to October 30th, 2012) were retrieved for the unscreened data set (Additional file 8: Appendix S3). Next, Bayesian analyses were performed using the unscreened data set. The results of Bayesian analyses are provided in Additional file 9: Appendix S4. DNA sequences that had been previously published were included directly in the data set, while the unpublished sequences could only be included if they clustered with reliable DNA sequences (including published and PCR-amplified ones), which belonged to the same species in the Bayesian phylogenetic analyses. As illustrated in Figure 1B, JQ625698 (unpublished sequence designated *Dermacentor marginatus*) was included in the data set, as it clustered with FN394329 (published sequence designated *D. marginatus*) in the Bayesian tree based on COI, while HM193887 (unpublished sequence designated *D. marginatus*) was excluded as it clustered with sample #65 (COI amplified from *D. nuttalli*). Overall, 430 unpublished sequences were screened and included in the data sets.

### Genetic divergence

**Figure 2:**
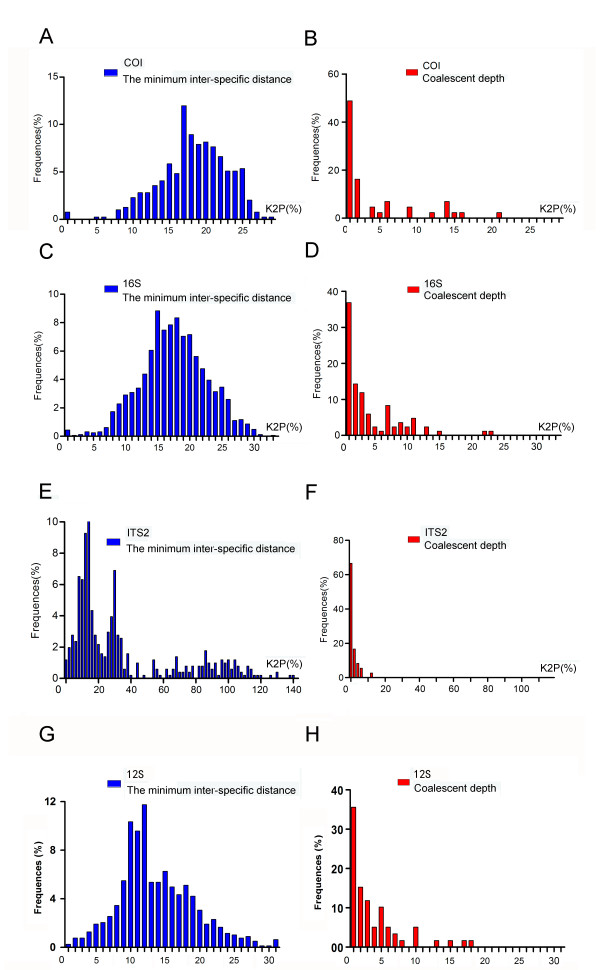
**Frequency distributions of K2P distances of COI**, **16S rDNA**, **ITS2 and 12S rDNA within and among tick species.** Panels show the distributions of minimum inter-specific K2P distances **(A, C, E, G)** and of coalescent depths **(B, D, F, H)** for COI **(A, B)**, 16S rDNA **(c, d)**, ITS2 **(E, F)** and 12S rDNA **(G, H)**.

Genetic divergence within and between species was calculated for data set 1. The average intra-specific distance, theta, and coalescent depth were calculated to determine the intra-specific variation using a K2P distance matrix. Average inter-specific distance, theta prime, and smallest inter-specific distances were used to characterize inter-specific divergence. When the four DNA barcoding regions were compared for their utility in tick species identification, the ITS2 region gave the highest value for all of the three inter-specific divergence parameters, although the standard deviations of these parameters were large (Table 1). Figure 2E shows that the distribution of the minimum inter-specific K2P distances for ITS2 was wide (0–140%), while the distributions for COI (Figure 2A), 16S rDNA (Figure 2C), and 12S rDNA (Figure 2G) were relatively narrowly (0–31%). ITS2 also exhibited the lowest intra-specific divergence according to the three inter-specific parameters (average inter-specific distance, theta prime, and smallest inter-specific distances) (Table 1). Figure 2F shows that the coalescent depth of ITS2 is narrowly distributed (0–11%), while those of COI (Figure 2B), 16S rDNA (Figure 2D), and 12S rDNA (Figure 2H) are more widely dispersed (0–23%). Moreover, the ratio of mean minimum inter-specific K2P distance for ITS2 (35.1%) to its coalescent depth (2.1%) was about 16.7, while it ranged from 3.9 to 4.6 for COI, 16S rDNA and 12S rDNA.

**Table 1:** **Inter**- **and intra**-**specific distances of candidate DNA markers of ticks**

**Markers**	**COI**	**16S**	**ITS2**	**12S**
**Average inter**-**specific distance**	0.174 ± 0.052	0.144 ± 0.059	0.417 ± 0.367	0.147 ± 0.049
**Theta prime**	0.185 ± 0.044	0.179 ± 0.050	0.359 ± 0.335	0.141 ± 0.055
**Minimum inter**-**specific distance**	0.178 ± 0.047	0.170 ± 0.051	0.351 ± 0.326	0.132 ± 0.055
**Average intra**-**specific distance**	0.014 ± 0.027	0.020 ± 0.033	0.003 ± 0.006	0.018 ± 0.026
**Theta**	0.017 ± 0.024	0.018 ± 0.027	0.012 ± 0.023	0.022 ± 0.030
**Coalescent depth**	0.038 ± 0.054	0.037 ± 0.047	0.021 ± 0.028	0.034 ± 0.030

All of the four gene regions investigated herein showed overlap between within-species and among-species K2P values using data set 1 (Figure 2). To determine whether the overlap was introduced by unpublished sequences or juvenile specimens collected in this study, we analysed the intra- and inter-specific distances using data set 3, which only includes 256 sequences obtained from the 68 adult specimens and 1834 published sequences from BOLD and GenBank. Additional file 10: Figure S1 shows that the overlaps still existed when the unpublished sequences or juvenile specimens were excluded from the analysis. Further analysis demonstrated that the overlaps were caused by the fact that some species were genetically closely related and some species could be divided into several genetically distant populations. Consequently, these species had inter-specific distances that were lower than the intra-specific distances. For example, the genetic distances between *Rhipicephalus microplus* and *R. annulatus* for COI, 16S rDNA, ITS2 and 12S rDNA were 6.0%, 1.2%, 1.1% and 1.0%, respectively, while the intra-specific distances for COI, 16S rDNA, ITS2 and 12S rDNA were 8.3% (*Ixodes lemuris*), 7.1% (*I. granulatus*), 5.8% (*I. granulatus*) and 9.3% (*I. granulatus*), respectively.

### Species boundaries

The distributions of false-positives and false-negatives calculated for ITS2 and 12S rDNA were shown in Additional file 11: Figure S2. For 12S rDNA, the sum of errors was minimized for the threshold value of 2.85%, whereas the threshold value of ITS2 was 2.27%. For 16S rDNA and COI, the values of the species boundaries were 5.25% and 6.13%, respectively, which is consistent with a previous study [12].

### Performances of the four DNA fragments to identify tick species

The DNA markers were evaluated by their “sequence identification success rates”. Data set 2 was utilized as the query data set, and data set 1 was used as the reference data set. The reliability of several methods of species identification (BLASTn, NN and tree-based methods) has been tested via simulated or empirical data sets in a previous study, and the results showed that BLASTn, distance, and liberal tree-based methods are likely to be equally successful [35]. In this study, we evaluated the efficiencies of four approaches to identify tick species: NN, BLASTn, the liberal tree-based method, and the liberal tree-based method (+ threshold).

For NN approach, all of the four DNA markers gave high sequence identification success rates. The highest sequence identification rate was given by COI (98.18%), followed by 12S rDNA (97.91%), 16S rDNA (97.44%) and ITS2 (96.19%), (Table 2). For BLASTn approach, the restrictive E-value cut-off (10^−6^) was used following a previous study [35]. The query’s ID was certain when the best hit (highest bit score) had an E-value below this cut-off. In the BLASTn analyses, all of the four DNA markers performed the same as in the NN method. The highest sequence identification rate was given by 12S rDNA (98.17%), followed by COI (97.95%), ITS2 (96.70%) and 16S rDNA (95.85%) (Table 2). In contrast, the liberal tree-based approach yielded lower sequence identification success rates than NN and BLASTn, ranging from 90.49% (16S rDNA) to 94.92% (ITS2) (Table 2). The liberal tree-based method (+threshold) had lower sequence identification success rates that ranged from 89.27% (16S rDNA) to 94.42% (ITS2), compared with the tree-based method without threshold (Table 2).

**Table 2:** Performance of four DNA fragments used for species identification of ticks based on their sequences identification success rates using four different methods

**Gene**	**No. of sequences**	**NN**		**BLASTn**		**Liberal tree**-**based**		**Liberal tree**-**based (+threshold)**	
		**Identified sequences**	**Rates**	**Identified sequences**	**Rates**	**Identified sequences**	**Rates**	**Identified sequences**	**Rates**
**COI**	439	431	98.18%	430	97.95%	409	93.17%	403	91.80%
**16S**	820	799	97.44%	786	95.85%	742	90.49%	732	89.27%
**ITS2**	394	379	96.19%	381	96.70%	374	94.92%	372	94.42%
**12S**	765	749	97.91%	751	98.17%	706	92.29%	698	91.24%

Based on the sequence identification success rates, we evaluated the influence of the DNA markers and the methods on species identification of ticks through Duncan’s multiple range tests. The results showed that there were no significant differences between the efficiencies of the four DNA markers to identify tick species (Table 3). As for the methods, BLASTn and NN significantly outperformed liberal tree-based methods (+ threshold or not) (Table 4), while no significant differences were found between BLASTn and NN (Table 4).

**Table 3:** Statistical analyses to evaluate how the DNA fragments influenced correct species identification success rates

**DNA fragments**	**COI**	**16S rDNA**	**ITS2**	**12S rDNA**
Rates of species identification success (±SE)	0.9527 ± 0.0327^a^	0.9326 ± 0.0399^b^	0.9556 ± 0.0106^c^	0.9490 ± 0.0365^d^

In the Duncan’s multiple range test, columns with different superscripted letters (a, b, c and d) indicate significant difference at P=0.05 between mean values; no significant difference was seen between mean values with the same superscript.

**Table 4:** Statistical analyses to evaluate the influence of methods on species identification success rates

**Methods**	**NN**	**BLASTn**	**Liberal tree**-**based**	**Liberal tree**-**based (+threshold**)
Rates of species identification success (±SE)	0.9743 ± 0.0088^a^	0.9716 ± 0.0109^a^	0.9271 ± 0.0184^b^	0.9168 ± 0.0212^b^

In the Duncan’s multiple range test, columns with different superscripted letters (a, b, c and d) indicate significant difference at P=0.05 between mean values; no significant difference was seen between mean values with the same superscript.

## Discussion

### Constructing reliable data sets for four DNA markers of ticks

Accurate assessment of a specific DNA region for species delimitation relies on the availability of sequences from vouchered specimens (i.e., BOLD: catalogue number and institution storing) identified by experts as being a particular species. We collected 84 tick specimens and identified them to species using a combination of morphological and molecular data. In addition, tick sequences from BOLD that had been published and supported by vouchers were included in our data set [36]. Published phylogenetic studies on ticks also provided reliable COI, 16S rDNA, ITS2 and 12S rDNA sequences via GenBank for our study, because those sequences originated from specimens identified by experienced tick experts [3,16,19,21,22]. Additionally, there is a large number of unpublished COI, 16S rDNA, ITS2 and 12S rDNA tick sequences in GenBank. To screen reliable sequences from these unpublished sequences, Bayesian analyses were performed as previously described [37]. Finally, 430 unpublished sequences were included in our data set.

### DNA barcodes may fail to identify some tick species because of taxonomic problems

Our analyses showed that genetic divergence in all of the four DNA markers between some congeneric species was very low (<0.5%). These results indicated that some species could not be distinguished from each other based on the four DNA fragments. For example, *Rhipicephalus sanguineus* could not be distinguished from *R. turanicus*, while *R. microplus* and *R. annulatus* proved difficult to distinguish. With regard to *R. sanguineus* and *R. turanicus*, the problem of species identification may be resolved by taxonomists and molecular biologists. Recently, a study pointed out the existence of different species under the names ‘*R. sanguineus*’ and ‘*R. turanicus*’ [4]. If this is true, the low resolving power of the DNA markers for species discrimination between ‘*R. sanguineus*’ and ‘*R. turanicus*’ might be caused by taxonomic problems. Therefore, it is important for taxonomists and molecular biologists to consider reclassification of the different populations currently designated ‘*R. sanguineus*’ and ‘*R. turanicus*’ so that it can be determined if different species exist within these populations.

### Primer performance

The 5’ region of COI is generally recognised as the standard barcoding marker for all animals. However, attempts to sequence COI in some species often fail because its sequence tends to be highly variable [13,14]. Ixodida consists of nearly 900 species and our multiple alignments of COI sequences of ticks exhibited high levels of nucleotide variability in the universal priming sites. This suggests that the universal primer pair LCO1490/HCO2198 would perhaps not be efficient in amplifying COI of ticks. To resolve this problem, a primer pair COI-F/COI-R was designed for ticks based on the universal barcoding primer pair LCO1490/HCO2198 [12]. Degenerate sites were included in the primers to ensure their suitability for different tick species.

There are currently several sets of primer pairs for amplification of COI from ticks (Additional file 2: Table S2). But which is the most efficient in recovering COI sequences across species is unknown. In this study, we evaluated the effectiveness of the above primers to recover COI sequences from 84 tick specimens. Results showed that Cox1F/Cox1R was the most successful (94.0%), while the primer pair (COI-F/COI-R) showed the second highest success rate (78.6%). Thus Cox1F/Cox1R and COI-F/COI-R primers offer distinct advantages over the other three primer sets. Furthermore, in this study, Cox1F/Cox1R successfully amplified 79 out of 84 tick specimens while COI-F/COI-R amplified 66 tick specimens (Additional file 2: Table S2). BJ116 was the only sample from which COI was not amplified by Cox1F/Cox1R but was amplified by COI-F/COI-R. Overall, we acquired 80 COI sequences from 84 tick specimens. Based on the above results, Cox1F/Cox1R should be the first choice for amplifying COI from ticks, while COI-F/COI-R could be used when Cox1F/Cox1R fails. Utilization of several COI primer sets would undoubtedly increase the amplification success rate for ticks.

It would be useful to know whether there are some tick species whose COI, 16S rDNA, ITS2 and 12S rDNA sequences are difficult to amplify. Consequently, we carefully analysed the tick specimens whose DNA sequences failed to be amplified. As shown in Additional file 1: Table S1, there were no circumstances where all of the four sequences were not amplified in a particular tick specimen. However, among the 84 tick specimens, there were two samples (XJ076 and BJ104) from which only 16S rDNA could be amplified. XJ076 and BJ104 were separately identified as *Hy. anatolicum* and *Haemaphysalis longicornis* (*Ha. longicornis*). Given that all of the four DNA fragments of the other *Hy. anatolicum* samples (XJ187, XJ190) and *Ha. longicornis* samples (SD109, BJ116) could be amplified, we believe that it was the quality of extracted DNA but not the species themselves that influenced the amplification success rate. PCR failures occurred for 12S rDNA from five out of 20 *D. nuttalli*, ITS2 from four out of 23 *Hy. asiaticum*, and ITS2 from three out of five *Hy. anatolicum* (Additional file 7: Table S5). Evidently, these DNA sequences were difficult to amplify from these particular species.

### Comparison of the four DNA fragments

In our previous study a DNA barcoding system for ticks based on three DNA markers (COI, 16S rDNA, and 18S rDNA) was developed. Although the DNA barcoding system could successfully identify tick species, utilization of three DNA fragments consequently increased the time, effort and expense of species identification. To resolve this problem a system for species identification of ticks based on a single molecular marker needs to be developed.

It is largely accepted that the accuracy of species delineation depends on the extent of, and separation between, intra-specific variation and inter-specific divergence in the selected marker. Our results showed that ITS2 possessed the greatest inter-specific divergence and minimum intra-specific variation among the four DNA fragments. The separation between intra-specific variation and inter-specific divergence of ITS2 is highest in the four loci, while separation of 12S rDNA is the lowest. Therefore, ITS2 should be the most efficient DNA marker for species discrimination of ticks. But the analyses of species identification success rates and Duncan’s multiple range tests showed that ITS2 has no advantage over COI, 16S rDNA and 12S rDNA in its resolution power for tick species identification. In other words, there are no statistically significant differences between the species identification efficiencies of the four DNA fragments (Table 3).

To explain the apparent inconsistency between the results of the genetic divergence analyses and the species identification success rates, we examined the genetic divergence of ITS2 deeply. We found that these species, which are genetically closely related or could be divided into several genetically distant populations were responsible for the poor performance of ITS2 in species discrimination. For example, the genetic distance of ITS2 between *R. microplus* and *R. annulatus* was 0.8% while some intra-specific distances for ITS2 within *I. granulatus* were 7.0%. Furthermore, we found that ITS2 displayed higher genetic divergence for species of ticks that were genetically distant but not for species that were closely related to each other, when compared with COI, 12S rDNA and 16S rDNA. For example, the theta prime (one parameter of inter-specific divergence) for ITS2 between *Ixodes ricinus* and *I. granulatus* (37.6%) is far higher than for COI (17.3%), 12S rDNA (13.8%) and 16S rDNA (9.5%). This explained why ITS2 had the greatest inter-specific divergence among the four DNA fragments. Based on above findings, it appears that ITS2 is suitable for species identification of genus *Ixodes* but not genus *Rhipicephalus*. To confirm this, we tested the reliability of ITS2 for species identification of ticks from *Rhipicephalus* and *Ixodes* using NN analysis. Results showed that the success rate of ITS2 for species identification of *Ixodes* was 100% (115 out of 115), while 92.98% (53 out of 57) for *Rhipicephalus*. The success rate of ITS2 for *Ixodes* was higher, while the rate for *Rhipicephalus* was lower, than the average rate of ITS2 across all tick genera (96.19%, Table 2).

As well as having high resolution power for species discrimination, a suitable genetic marker for species identification needs to meet a number of important criteria. First, it must be flanked by conserved regions of DNA that can be used to develop universal primers and the primers need to have high amplification efficiencies and species-specificity. Second, the DNA fragments should be short and lack heterozygosity, so that they can be sequenced directly in a single reaction. Third, sequence alignments of such fragments should be easy to conduct and generate unambiguous results.

As to the first criteria (*i.e*., primer efficiency), in this study we found that 16S rDNA (97.6%) and COI (95.2%) were more reliable than ITS2 (84.5%) and 12S rDNA (88.1%) in terms of sequence recovery rates. Nevertheless, it cannot be ruled out that better primers could be designed to improve the recovery rates of 12S rDNA and ITS2 from ticks.

For the second criteria (i.e., sequence length), the lengths of 16S rDNA and 12S rDNA fragment are both less than 450 bp, making it easy to obtain accurate sequences in a single reaction. Although the length of COI fragment is about 670 bp, it is still possible to obtain the full length sequence in a single reaction. However, we found some low-quality base peaks at the end of the sequence reads and the quality of the data might be affected by this. In contrast, the length of ITS2 fragment exceeds 1000 bp, making two reactions necessary to sequence it completely. Hence, the quality of ITS2 sequences is likely to be the lowest among the four DNA fragments. As shown in Additional file 3: Appendix S1, the sequencing quality of 16S rDNA was highest among the four loci, followed by COI and 12S rDNA, while the quality of ITS2 was the lowest. These results are consistent with our expectations.

For the third criteria (i.e., sequence alignment), we found that the sequence alignment for COI was the most straightforward of the four DNA fragments because COI is a protein-coding sequence and there were no gaps within the alignment, while the ITS2 alignment was the most difficult because ITS2 sequences from different tick species are highly variable.

Considering all of the above aspects, at present, both 16S rDNA and COI appear to be suitable DNA markers for tick species identification compared with 12S rDNA and ITS2. However, it is difficult to determine whether COI is a more suitable DNA marker for tick species identification than 16S rDNA as both of them have advantages and disadvantages: COI sequences are easier to align than 16S rDNA, while the sequencing quality of 16S rDNA is higher than that of COI. Regardless, some other important factors should be considered. The 5’ region of COI is the standard marker for DNA barcoding and a large number of COI sequences from animals have been deposited in BOLD. As no other comparable database currently exists, it remains an important resource for COI barcoding as it contributes to and enables comparisons of standard gene fragments in the global dataset. From this aspect, COI has an advantage over 16S rDNA.

### Methods used for species identification of ticks

DNA sequences can be useful tools for species identification in most animals. To improve the success rates of species identification, several molecular genetic techniques based on single-gene sequence similarity or phylogenies have been developed and tested [35]. It has been suggested that BLASTn, NN and liberal tree-based methods are equally reliable and make more correct species identifications than strict tree-based methods. Our previous study showed that the NJ-tree based method could be utilized for species identification of ticks [12]. But it is still unknown whether other methods (especially BLASTn or NN) perform better than tree-based methods. In this study, we directly evaluated the reliability of four methods (BLAST, NN, liberal NJ-tree based method and liberal NJ-tree based method (+threshold)) for species identification of ticks using our tick data sets.

Our results showed that BLASTn and NN performed well and the identification success rates of the four DNA fragments ranged from 95.85% to 98.18%. In addition, Duncan’s multiple range tests illustrated that BLASTn and NN performed equally as well as each other and significantly outperformed liberal tree-based methods (+ threshold or not) in tick species identification (Table 4). Evidently, to correctly identify sequences from the same species using a tree-based method requires the sequences to form a monospecific clade. Otherwise, some sequences (and sometimes all the sequences) will not be assigned to the correct species. In contrast, in the present study, when faced with a similar situation, BLASTn and NN both avoided this disadvantage and only a few of the sequences were assigned to incorrect taxonomies. In addition, alignments of DNA sequences are necessary for NN but not BLASTn. Therefore, BLASTn has an advantage over NN as conducting sequence alignments requires time and effort.

## Conclusions

As the standard DNA barcode, COI is the first choice for species identification of ticks, while 16S rDNA, ITS2 and 12S rDNA could be used as complementary to COI, thereby circumventing situations where COI fails to produce reliable results. Moreover, either NN or BLASTn could be used for tick species identification because both methods outperformed tree-based methods (+threshold or otherwise).

## Abbreviations

K2P: Kimura 2-parameter genetic distances; NJ tree: Neighbour-joining phylogenetic tree; COI: cytochrome *c* oxidase I; 16S rDNA: 16S ribosomal DNA; 12S rDNA: 12S ribosomal DNA; NN: nearest neighbour.

## Competing interests

The authors declare that they have no competing interests.

## Authors’ contributions

JZL, SQW carried out the molecular genetic studies, participated in the sequence alignment and drafted the manuscript. YC and YNZ collected tick specimens and carried out the morphological identification of samples. CYF, XFY and JHD participated in the sequence alignment and the design of the study. GLJ, CXW, QW and LM performed the statistical analyses. XML conceived of this study, and participated in its design and coordination and helped to draft the manuscript. All authors read and approved the final manuscript.

## Supplementary Material

Additional file 1: Table S1 Summary of the identification results of tick specimens. Click here for file

Additional file 2: Table S2 COI, 16S rDNA, ITS2 and 12S rDNA primer pairs employed in this study. Click here for file

Additional file 3 **Appendix S1.** Read length and quality scores for each unidirectional and bidirectional sequence. Click here for file

Additional file 4: Table S3 Summary information on COI, 16S rDNA, ITS2 and 12S rDNA for data set 1. Unpublished sequences were screened by Bayesian analyses as described in the Results section. Click here for file

Additional file 5 **Appendix S2.** The data sets 1 to 3 utilized in this manuscript. Click here for file

Additional file 6: Table S4 The performance of different primer pairs in amplifying COI from 84 tick specimens. Click here for file

Additional file 7: Table S5 Summary information on the DNA sequences that failed to be PCR amplified from the 84 tick specimens collected in this study. Click here for file

Additional file 8 **Appendix S3.** The unscreened data set. Click here for file

Additional file 9 **Appendix S4.** The results of Bayesian analyses. Click here for file

Additional file 10: Figure S1 Frequency distributions of K2P for COI, 16S rDNA, ITS2 and 12S rDNA within and among species based on data set 3. Click here for file

Additional file 11: Figure S2 Cumulative error distributions among the species of ticks calculated from (A) 12S and (B) ITS2. Click here for file
