# Supplementary material for: Assessment of four DNA fragments (COI, 16S rDNA, ITS2, 12S rDNA) for species identification of the Ixodida (Acari: Ixodida)
Source: Parasit Vectors. 2014 Mar 3;7:93. doi: 10.1186/1756-3305-7-93 (PMC3945964; doi:10.1186/1756-3305-7-93)
Supplement: Additional file 1: Table S1 — Summary of the identification results of tick specimens. [file 1756-3305-7-93-S1.doc]

## Table S1 - Summary of the identification results of tick specimens.

| Specimens  code | COI | 16S rRNA | ITS2 | 12S rDNA | Identification  results | Developmental stage | Collection localities |
| --- | --- | --- | --- | --- | --- | --- | --- |
| YN01 | JX051119 | JX051062 | KC203364 | KF583582 | *Rhipicephalus microplus* | Adult | Kunming |
| YN02 | JX051120 | JX051063 | KC203365 | No sequence | *Rhipicephalus microplus* | Adult | Kunming |
| YN07 | JX051121 | JX051064 | KC203395 | KF583637 | *Haemaphysalis longicornis* | Adult | Kunming |
| YN08 | JX051122 | JX051065 | KC203396 | KF583639 | *Haemaphysalis longicornis* | Adult | Kunming |
| YN09 | JX051123 | JX051066 | No sequence | KF583589 | *Haemaphysalis longicornis* | Adult | Kunming |
| YN010 | KF583579 | No sequence | KC203366 | KF583583 | *Rhipicephalus microplus* | Adult | Kunming |
| YN011 | JX051124 | JX051067 | KC203397 | KF583584 | *Haemaphysalis longicornis* | Adult | Kunming |
| YN012 | JX051125 | JX051068 | KC203367 | KF583585 | *Rhipicephalus microplus* | Adult | Kunming |
| YN013 | JX051126 | JX051069 | KC203398 | KF583586 | *Haemaphysalis longicornis* | Adult | Kunming |
| YN014 | JX051127 | JX051070 | KC203399 | KF583587 | *Haemaphysalis longicornis* | Adult | Kunming |
| YN015 | JX051128 | JX051071 | KC203400 | KF583588 | *Haemaphysalis longicornis* | Adult | Kunming |
| YN016 | JX051129 | JX051072 | KC203368 | KF583590 | *Rhipicephalus microplus* | Adult | Kunming |
| YN017 | JX051130 | JX051073 | KC203401 | KF583592 | *Haemaphysalis longicornis* | Adult | Kunming |
| IM034 | JX051131 | JX051074 | KC203371 | KF583593 | *Hyalomma asiaticum* | Adult | Ordos |
| IM035 | JX051132 | JX051075 | KC203372 | KF583594 | *Hyalomma asiaticum* | Adult | Ordos |
| IM036 | JX051133 | JX051076 | KC203373 | KF583595 | *Hyalomma asiaticum* | Adult | Ordos |
| IM037 | JX051134 | JX051077 | No sequence | KF583596 | *Hyalomma asiaticum* | Adult | Ordos |
| IM038 | JX051135 | JX051078 | KC203374 | KF583597 | *Hyalomma asiaticum* | Adult | Ordos |
| IM039 | JX051136 | JX051079 | KC203375 | KF583620 | *Hyalomma asiaticum* | Adult | Ordos |
| IM041 | JX051137 | JX051080 | KC203376 | KF583621 | *Hyalomma asiaticum* | Adult | Ordos |
| IM042 | JX051138 | JX051081 | No sequence | KF583622 | *Hyalomma asiaticum* | Adult | Ordos |
| IM043 | JX051139 | JX051082 | No sequence | KF583623 | *Hyalomma asiaticum* | Adult | Ordos |
| IM044 | JX051140 | JX051083 | KC203377 | KF583624 | *Hyalomma asiaticum* | Adult | Ordos |
| IM045 | JX051141 | JX051084 | KC203378 | KF583625 | *Hyalomma asiaticum* | Adult | Ordos |
| IM046 | JX051142 | JX051085 | No sequence | KF583626 | *Hyalomma asiaticum* | Adult | Ordos |
| IM047 | JX051143 | JX051086 | KC203379 | KF583627 | *Hyalomma asiaticum* | Adult | Ordos |
| IM048 | JX051144 | JX051087 | KC203380 | KF583628 | *Hyalomma asiaticum* | Adult | Ordos |
| IM049 | JX051145 | JX051088 | KC203381 | KF583629 | *Hyalomma asiaticum* | Adult | Ordos |
| IM050 | JX051146 | JX051089 | KC203382 | KF583630 | *Hyalomma asiaticum* | Adult | Ordos |
| IM051 | JX051147 | JX051090 | KC203383 | KF583631 | *Hyalomma asiaticum* | Adult | Ordos |
| IM053 | JX051148 | JX051091 | KC203384 | KF583633 | *Hyalomma asiaticum* | Adult | Ordos |
| IM054 | JX051149 | JX051092 | KC203385 | KF583634 | *Hyalomma asiaticum* | Adult | Ordos |
| IM055 | JX051150 | JX051093 | KC203386 | KF583635 | *Hyalomma asiaticum* | Adult | Ordos |
| XJ056 | JX051151 | JX051094 | KC203415 | KF583640 | *Dermacentor marginatus* | Adult | Chaxian |
| XJ057 | KF583568 | JX051095 | No sequence | KF583641 | *Dermacentor marginatus* | Adult | Chaxian |
| XJ058 | JX051152 | JX051096 | KC203417 | KF583642 | *Dermacentor marginatus* | Adult | Chaxian |
| XJ059 | KF583569 | JX051097 | KC203416 | KF583643 | *Dermacentor marginatus* | Adult | Chaxian |
| XJ060 | JX051153 | JX051098 | No sequence | KF583644 | *Dermacentor marginatus* | Adult | Chaxian |
| XJ061 | KF583570 | JX051099 | KC203418 | KF583645 | *Dermacentor nuttalli* | Adult | Hejing |
| XJ062 | JX051154 | JX051100 | KC203419 | KF583632 | *Dermacentor nuttalli* | Adult | Hejing |
| XJ063 | KF583576 | JX051101 | No sequence | No sequence | *Hyalomma anatolicum* | Nymph | Kashi |
| XJ065 | JX051155 | JX051102 | KC203420 | KF583646 | *Dermacentor nuttalli* | Larva | Hejing |
| XJ066 | KF583571 | JX051103 | KC203421 | No sequence | *Dermacentor nuttalli* | Larva | Hejing |
| XJ067 | JX051156 | JX051104 | KC203422 | KF583647 | *Dermacentor nuttalli* | Adult | Hejing |
| XJ068 | JX051157 | JX051105 | KC203423 | KF583648 | *Dermacentor nuttalli* | Adult | Hejing |
| XJ071 | JX051158 | JX051106 | KC203424 | No sequence | *Dermacentor nuttalli* | Larva | Hejing |
| XJ072 | JX051159 | JX051107 | KC203425 | No sequence | *Dermacentor nuttalli* | Larva | Hejing |
| XJ074 | KF583577 | JX051108 | No sequence | KF583638 | *Hyalomma anatolicum* | Nymph | Kashi |
| XJ076 | No sequence | JX051109 | No sequence | No sequence | *Hyalomma anatolicum* | Nymph | Kashi |
| XJ083 | No sequence | JX051110 | KC203426 | KF583649 | *Dermacentor nuttalli* | Larva | Hejing |
| XJ084 | JX051160 | JX051111 | KC203427 | KF583650 | *Dermacentor nuttalli* | Larva | Hejing |
| XJ085 | JX051161 | JX051112 | No sequence | KF583651 | *Dermacentor nuttalli* | Larva | Hejing |
| XJ087 | JX051162 | JX051113 | KC203428 | KF583652 | *Dermacentor nuttalli* | Larva | Hejing |
| XJ088 | JX051163 | JX051114 | KC203429 | KF583653 | *Dermacentor nuttalli* | Larva | Hejing |
| XJ089 | JX051164 | JX051115 | KC203430 | KF583654 | *Dermacentor nuttalli* | Larva | Hejing |
| XJ090 | KF583574 | JX051116 | KC203431 | No sequence | *Dermacentor nuttalli* | Larva | Hejing |
| XJ091 | KF583573 | JX051117 | KC203432 | KF583655 | *Dermacentor nuttalli* | Larva | Hejing |
| XJ092 | KF583572 | JX051118 | KC203433 | No sequence | *Dermacentor nuttalli* | Larva | Hejing |
| BJ095 | KC203441 | KC203352 | KC203402 | KF583636 | *Haemaphysalis longicornis* | Adult | Fangshan |
| BJ101 | KC203442 | KC203341 | KC203403 | KF583598 | *Haemaphysalis longicornis* | Adult | Fangshan |
| BJ104 | No sequence | KC203353 | No sequence | No sequence | *Haemaphysalis longicornis* | Adult | Fangshan |
| SD109 | KC203443 | KC203354 | KC203404 | KF583599 | *Haemaphysalis longicornis* | Adult | Rizhao |
| BJ111 | KC203444 | KC203355 | KC203405 | KF583600 | *Haemaphysalis longicornis* | Adult | Fangshan |
| BJ114 | KC203445 | KC203356 | KC203406 | KF583601 | *Haemaphysalis longicornis* | Adult | Fangshan |
| BJ116 | KC203446 | KC203357 | KC203407 | KF583602 | *Haemaphysalis longicornis* | Adult | Fangshan |
| BJ120 | KC203447 | KC203358 | KC203408 | KF583603 | *Haemaphysalis longicornis* | Adult | Fangshan |
| XJ142 | KF583580 | KC203346 | KC203390 | KF583604 | *Hyalomma detritum* | Adult | Tulufan |
| XJ143 | KC203434 | No sequence | KC203393 | No sequence | *Hyalomma detritum* | Adult | Tulufan |
| XJ144 | KC203435 | KC203347 | KC203391 | KF583605 | *Hyalomma detritum* | Adult | Tulufan |
| XJ145 | KC203436 | KC203348 | KC203392 | KF583606 | *Hyalomma detritum* | Adult | Tulufan |
| XJ150 | KF583581 | KC203349 | KC203394 | KF583607 | *Hyalomma detritum* | Adult | Tulufan |
| SD153 | KC203448 | KC203359 | KC203409 | KF583608 | *Haemaphysalis longicornis* | Adult | Rizhao |
| SD154 | KF583575 | KC203360 | KC203410 | KF583609 | *Haemaphysalis longicornis* | Adult | Rizhao |
| SD160 | KC203449 | KC203361 | KC203411 | KF583610 | *Haemaphysalis longicornis* | Adult | Rizhao |
| BJ165 | KC203450 | KC203342 | KC203412 | KF583611 | *Haemaphysalis longicornis* | Adult | Fangshan |
| XJ179 | KC203451 | KC203343 | KC203413 | KF583612 | *Dermacentor nuttalli* | Adult | Tulufan |
| XJ185 | KC203452 | KC203344 | KC203414 | KF583613 | *Dermacentor nuttalli* | Adult | Tulufan |
| XJ186 | KC203453 | KC203345 | No sequence | KF583614 | *Dermacentor nuttalli* | Adult | Tulufan |
| XJ187 | KC203438 | KC203338 | KC203369 | KF583615 | *Hyalomma anatolicum* | Adult | Tulufan |
| XJ189 | KC203439 | KC203339 | KC203387 | KF583591 | *Hyalomma asiaticum* | Adult | Tulufan |
| XJ190 | KC203437 | KC203340 | KC203370 | KF583616 | *Hyalomma anatolicum* | Adult | Tulufan |
| XJ191 | KF583578 | KC203350 | KC203388 | KF583617 | *Hyalomma asiaticum* | Adult | Tulufan |
| XJ192 | KC203440 | KC203351 | KC203389 | KF583618 | *Hyalomma asiaticum* | Adult | Tulufan |
| HB193 | No sequence | KC203362 | KC203363 | KF583619 | [*Rhipicephalus sanguineus*](http://www.search.com/search?q=Rhipicephalus+sanguineus&respell=1) | Adult | Hebei |
