# Supplementary material for: Assessment of four DNA fragments (COI, 16S rDNA, ITS2, 12S rDNA) for species identification of the Ixodida (Acari: Ixodida)
Source: Parasit Vectors. 2014 Mar 3;7:93. doi: 10.1186/1756-3305-7-93 (PMC3945964; doi:10.1186/1756-3305-7-93)
Supplement: Additional file 2: Table S2 — COI, 16S rDNA, ITS2 and 12S rDNA primer pairs employed in this study. [file 1756-3305-7-93-S2.doc]

## Table S2 - COI, 16S rDNA, ITS2 and 12S rDNA primer pairs employed in this study.

| **Gene** | **Primer Pairs** | **Forward** | **Reverse** | **Length of fragments** | **Reference** |
| --- | --- | --- | --- | --- | --- |
| **COI** | COI-F/  COI-R | 5’ATCATAAAKAYHTTGG 3’ | 5’GGGTGACCRAARAAHCA 3’ | Approximately  680 bp |  |
|  | TY-J-1449/  C1-N-2312 | 5’AATTTACAGTTTATCGCCT3’ | 5’CATACAATAAAGCCTAATA3’ | Approximately  860 bp |  |
|  | Cox1F/  Cox1R | 5’GGAACAATATATTTAATTTTTGG3’ | 5’ATCTATCCCTACTGTAAATATATG3’ | Approximately  820 bp |  |
|  | HCO2064/  HCO1215 | 5’GGTGGGCTCATACAATAAATCC3’ | 5’GCCATTTTACCGCGATGA3’ | Approximately  860 bp |  |
|  | HCO1490/  LCO2198 | 5’GGTCAACAAATCATAAAGATATTGG3’ | 5’TAAACTTCAGGGTGACCAAAAATCA3’ | Approximately  680 bp |  |
| **16S** | 16S-F/  16S-R1/ | 5’TTAAATTGCTGTRGTATT3’ | 5’CCGGTCTGAACTCASAWC3’ | Approximately  455 bp |  |
| **ITS2** | ITS2-F/  ITS2-R | 5’ACATTGCGGCCTTGGGTCTT3’ | 5’TCGCCTGATCTGAGGTCGAC3’ | Ranged from 1200 to 1600 bp | This study |
| **12S** | T1B/T2A | 5’AAACTAGGATTAGATACCCT3’ | 5’AATGAGAGCGACGGGCGATGT3’ | Approximately  320 bp |  |
