# Supplementary material for: Assessment of four DNA fragments (COI, 16S rDNA, ITS2, 12S rDNA) for species identification of the Ixodida (Acari: Ixodida)
Source: Parasit Vectors. 2014 Mar 3;7:93. doi: 10.1186/1756-3305-7-93 (PMC3945964; doi:10.1186/1756-3305-7-93)
Supplement: Additional file 4: Table S3 — Summary information on COI, 16S rDNA, ITS2 and 12S rDNA for data set 1. Unpublished sequences were screened by Bayesian analyses as described in the Results section. [file 1756-3305-7-93-S4.doc]

## Table S3 - Summary information on COI, 16S rDNA, ITS2 and 12S rDNA for data set 1.

| **Markers** | **No. of all sequences** | **No. of PCR-amplified sequences** | **No. of published sequences** | **No. of unpublished sequences** |
| --- | --- | --- | --- | --- |
| **COI** | **465** | **80** | **325** | **60** |
| **16S** | **873** | **82** | **643** | **148** |
| **ITS2** | **427** | **71** | **320** | **36** |
| **12S** | **806** | **74** | **546** | **186** |

Unpublished sequences were screened by [Bayesian analyses](http://webpages.icav.up.pt/PTDC/BIA-BEC/104097/2008/23.pdf) as described in the Results section.
