# Supplementary material for: Assessment of four DNA fragments (COI, 16S rDNA, ITS2, 12S rDNA) for species identification of the Ixodida (Acari: Ixodida)
Source: Parasit Vectors. 2014 Mar 3;7:93. doi: 10.1186/1756-3305-7-93 (PMC3945964; doi:10.1186/1756-3305-7-93)
Supplement: Additional file 6: Table S4 — The performance of different primer pairs in amplifying COI from 84 tick specimens. [file 1756-3305-7-93-S6.doc]

## Table S4 - The performance of different primer pairs in amplifying COI from 84 tick specimens.

| **Primer pairs** | **COI-F/**  **COI-R** | **TY-J-1449/**  **C1-N-2312** | **Cox1F/**  **Cox1R** | **HCO2064/**  **HCO1215** | **HCO1490/**  **LCO2198** |
| --- | --- | --- | --- | --- | --- |
| **No. of obtained COI sequences** | **66** | **23** | **79** | **19** | **12** |
| **Success Rates** | **78.6%** | **27.4%** | **94.0%** | **22.6%** | **14.3%** |
