# Supplementary material for: Assessment of four DNA fragments (COI, 16S rDNA, ITS2, 12S rDNA) for species identification of the Ixodida (Acari: Ixodida)
Source: Parasit Vectors. 2014 Mar 3;7:93. doi: 10.1186/1756-3305-7-93 (PMC3945964; doi:10.1186/1756-3305-7-93)
Supplement: Additional file 7: Table S5 — Summary information on the DNA sequences that failed to be PCR amplified from the 84 tick specimens collected in this study. [file 1756-3305-7-93-S7.doc]

## Table S5 - Summary information on the DNA sequences that failed to be PCR amplified from the 84 tick specimens collected in this study.

| **DNA fragments** | **Species** | **No. of amplifications attempted in that species** | **No. of failed amplifications in that species** | **Rates of amplification failed** |
| --- | --- | --- | --- | --- |
| **COI** | ***Rhipicephalus sanguineus*** | **1** | **1** | **100.0%** |
|  | ***Haemaphysalis longicornis*** | **20** | **1** | **5.0%** |
|  | ***Hyalomma anatolicum*** | **5** | **1** | **20.0%** |
|  | ***Dermacentor nuttalli*** | **20** | **1** | **5.0%** |
| **16S rDNA** | ***Hyalomma detritum*** | **5** | **1** | **20.0%** |
|  | ***Rhipicephalus microplus*** | **5** | **1** | **20.0%** |
| **ITS2** | ***Hyalomma asiaticum*** | **23** | **4** | **17.4%** |
|  | ***Hyalomma anatolicum*** | **5** | **3** | **60.0%** |
|  | ***Dermacentor nuttalli*** | **20** | **2** | **10.0%** |
|  | ***Dermacentor marginatus*** | **5** | **2** | **40.0%** |
|  | ***Haemaphysalis longicornis*** | **20** | **2** | **10.0%** |
| **12S rDNA** | ***Dermacentor nuttalli*** | **20** | **5** | **25.0%** |
|  | ***Hyalomma anatolicum*** | **5** | **2** | **40.0%** |
|  | ***Rhipicephalus microplus*** | **5** | **1** | **20.0%** |
|  | ***Hyalomma detritum*** | **5** | **1** | **20.0%** |
|  | ***Haemaphysalis longicornis*** | **20** | **1** | **5.0%** |
