# Supplementary material for: Assessment of four DNA fragments (COI, 16S rDNA, ITS2, 12S rDNA) for species identification of the Ixodida (Acari: Ixodida)
Source: Parasit Vectors. 2014 Mar 3;7:93. doi: 10.1186/1756-3305-7-93 (PMC3945964; doi:10.1186/1756-3305-7-93)
Supplement: Additional file 9 — Appendix S4. The results of Bayesian analyses. [file 1756-3305-7-93-S9.zip › Appendix.S4/16S rDNA Bayesian analyses/Haemaphysalis 16S.pdf]

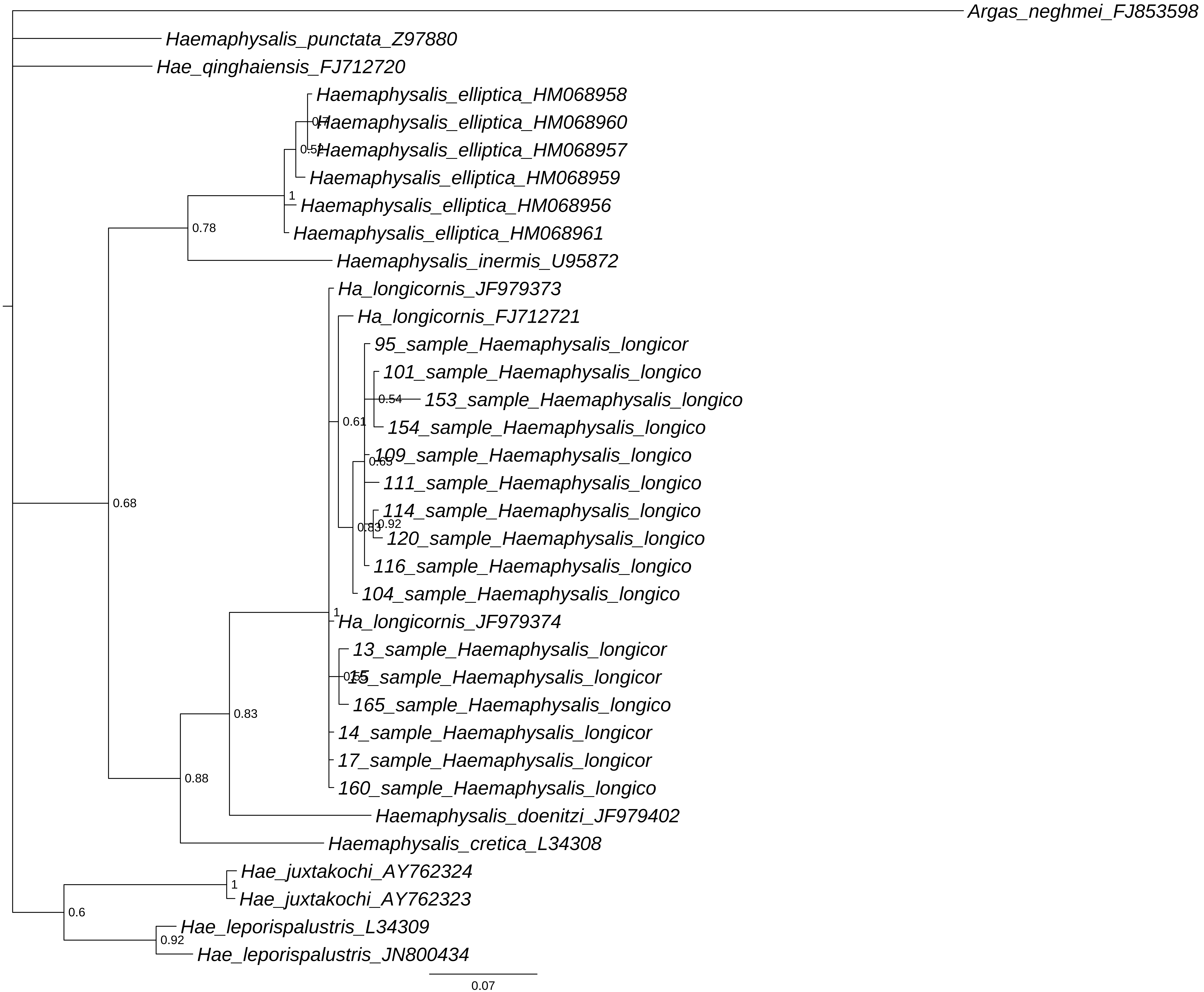
Argas_neghmei_FJ853598
Haemaphysalis_punctata_Z97880
Hae_qinghaiensis_FJ712720
Haemaphysalis_elliptica_HM068958
Haemaphysalis_elliptica_HM068960
Haemaphysalis_elliptica_HM068957
Haemaphysalis_elliptica_HM068959
Haemaphysalis_elliptica_HM068956
Haemaphysalis_elliptica_HM068961
Haemaphysalis_inermis_U95872
Ha_longicornis_JF979373
Ha_longicornis_FJ712721
95_sample_Haemaphysalis_longicor
101_sample_Haemaphysalis_longico
153_sample_Haemaphysalis_longico
154_sample_Haemaphysalis_longico
109_sample_Haemaphysalis_longico
111_sample_Haemaphysalis_longico
114_sample_Haemaphysalis_longico
120_sample_Haemaphysalis_longico
116_sample_Haemaphysalis_longico
104_sample_Haemaphysalis_longico
Ha_longicornis_JF979374
13_sample_Haemaphysalis_longicor
15_sample_Haemaphysalis_longicor
165_sample_Haemaphysalis_longico
14_sample_Haemaphysalis_longicor
17_sample_Haemaphysalis_longicor
160_sample_Haemaphysalis_longico
Haemaphysalis_doenitzi_JF979402
Haemaphysalis_cretica_L34308
Hae_juxtakochi_AY762324
Hae_juxtakochi_AY762323
Hae_leporispalustris_L34309
Hae_leporispalustris_JN800434
1
0.78
0.54
0.61
0.92
0.83
0.68
0.88
0.6
0.07
