# Supplementary figures and images for: Assessment of four DNA fragments (COI, 16S rDNA, ITS2, 12S rDNA) for species identification of the Ixodida (Acari: Ixodida)
Source: Parasit Vectors. 2014 Mar 3;7:93. doi: 10.1186/1756-3305-7-93 (PMC3945964; doi:10.1186/1756-3305-7-93)

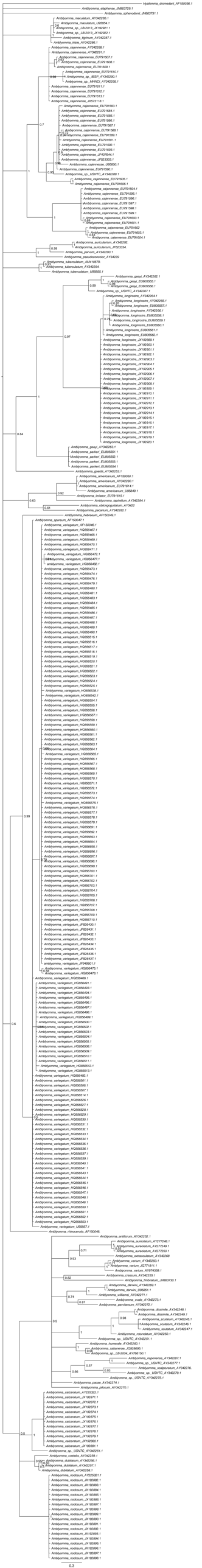

Supplement: Additional file 9 — Appendix S4. The results of Bayesian analyses. [file 1756-3305-7-93-S9.zip › Appendix.S4/12S Bayesian analyses/Amblyomma 12S.pdf]

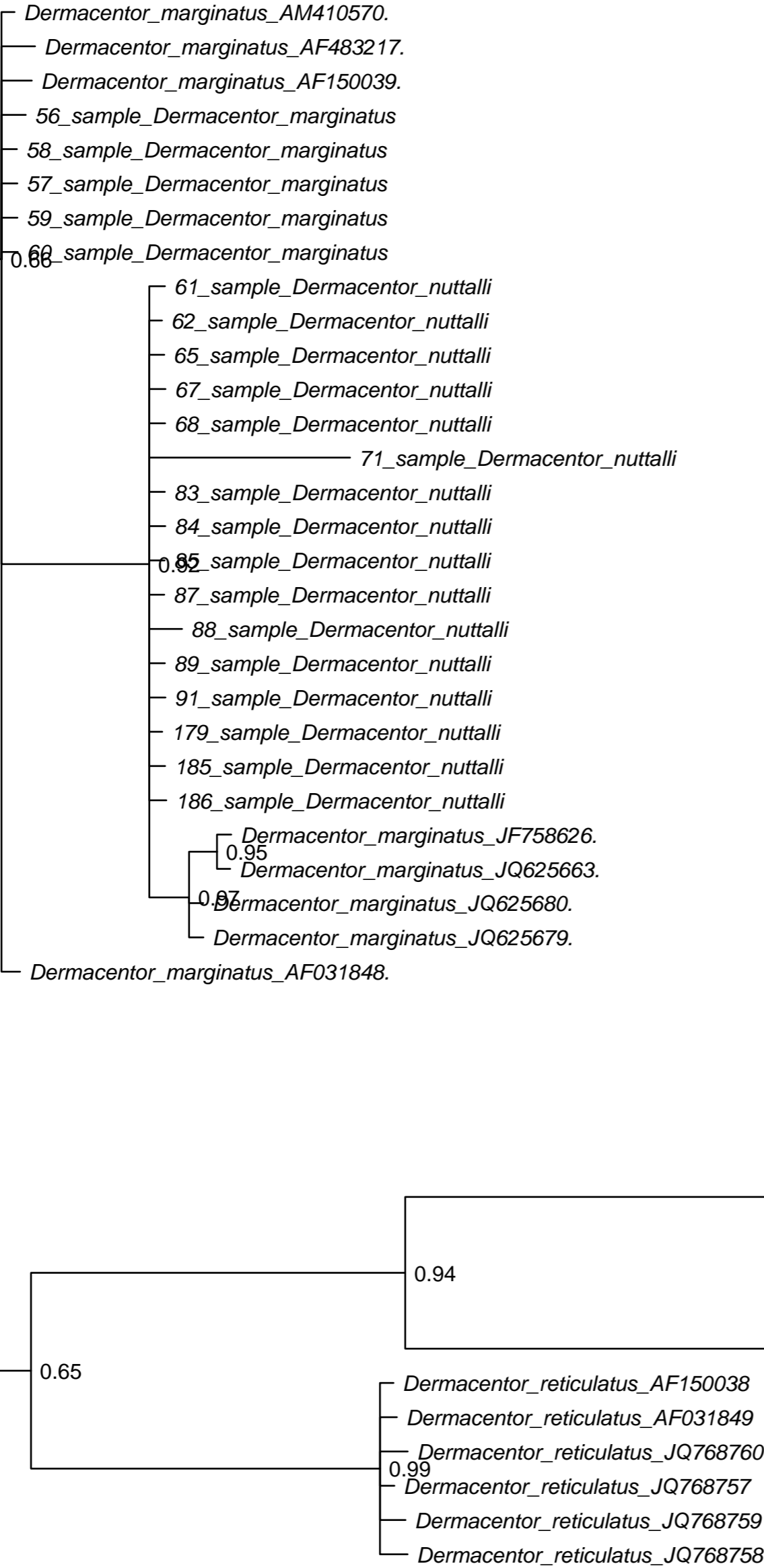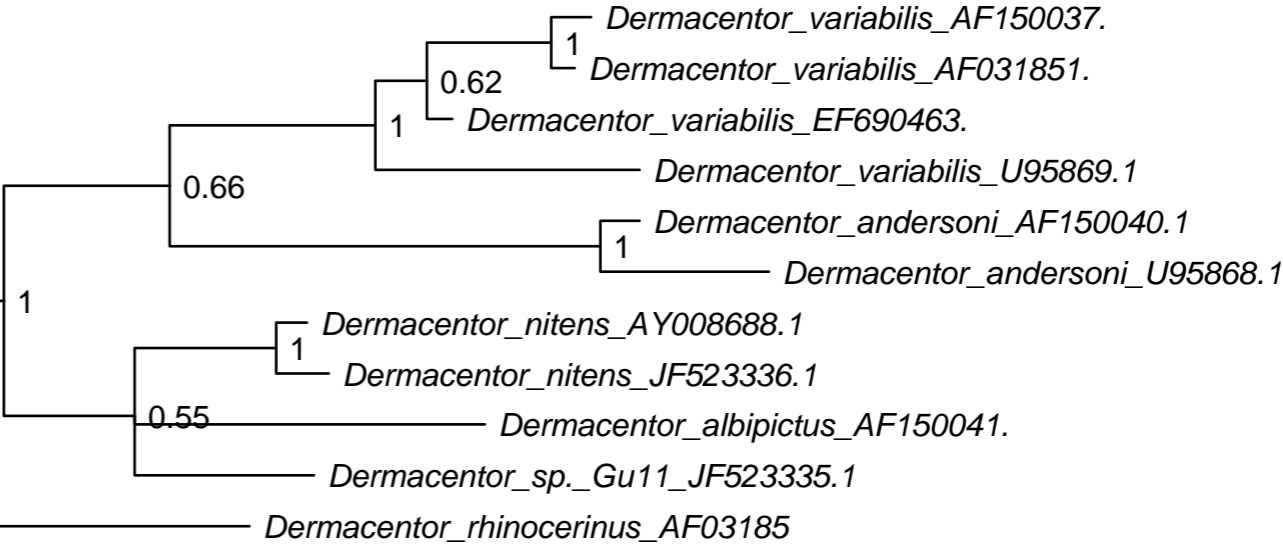

Supplement: Additional file 9 — Appendix S4. The results of Bayesian analyses. [file 1756-3305-7-93-S9.zip › Appendix.S4/12S Bayesian analyses/Dermacentor 12S.pdf]

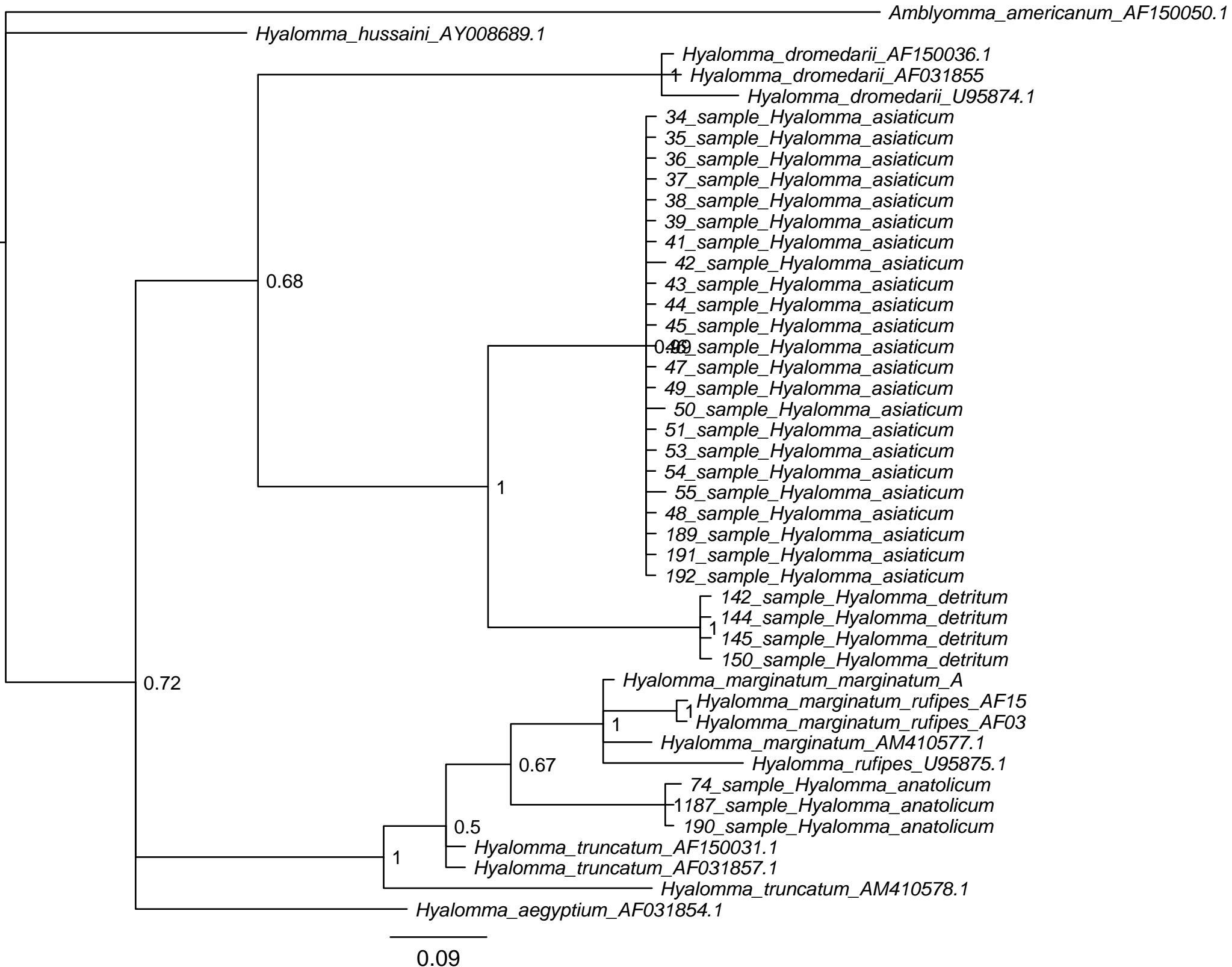

Supplement: Additional file 9 — Appendix S4. The results of Bayesian analyses. [file 1756-3305-7-93-S9.zip › Appendix.S4/12S Bayesian analyses/Hyalomma 12S.pdf]

1 Ixodes\_uriae\_U95907.1  
Ixodes\_uriae\_AM410581.1

Haemaphysalis\_parva

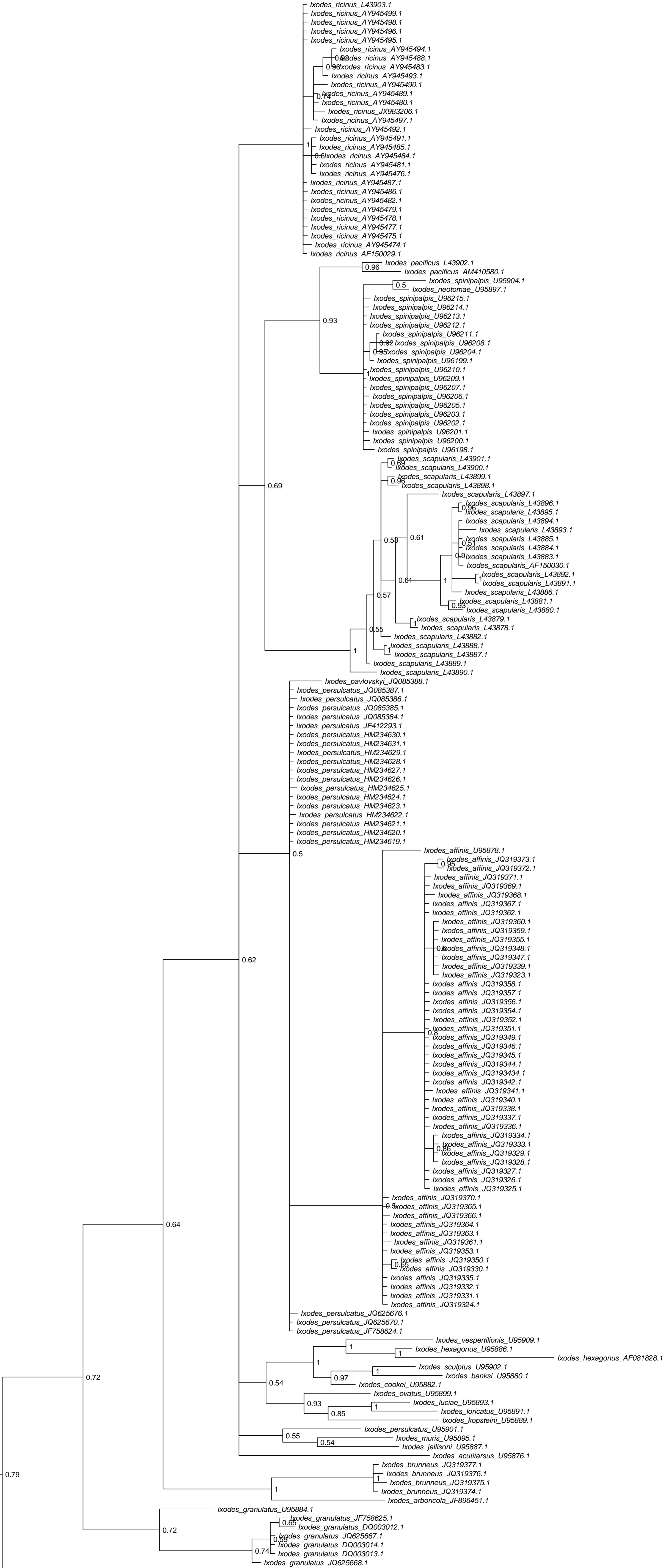

Supplement: Additional file 9 — Appendix S4. The results of Bayesian analyses. [file 1756-3305-7-93-S9.zip › Appendix.S4/12S Bayesian analyses/Ixodes 12S.pdf]

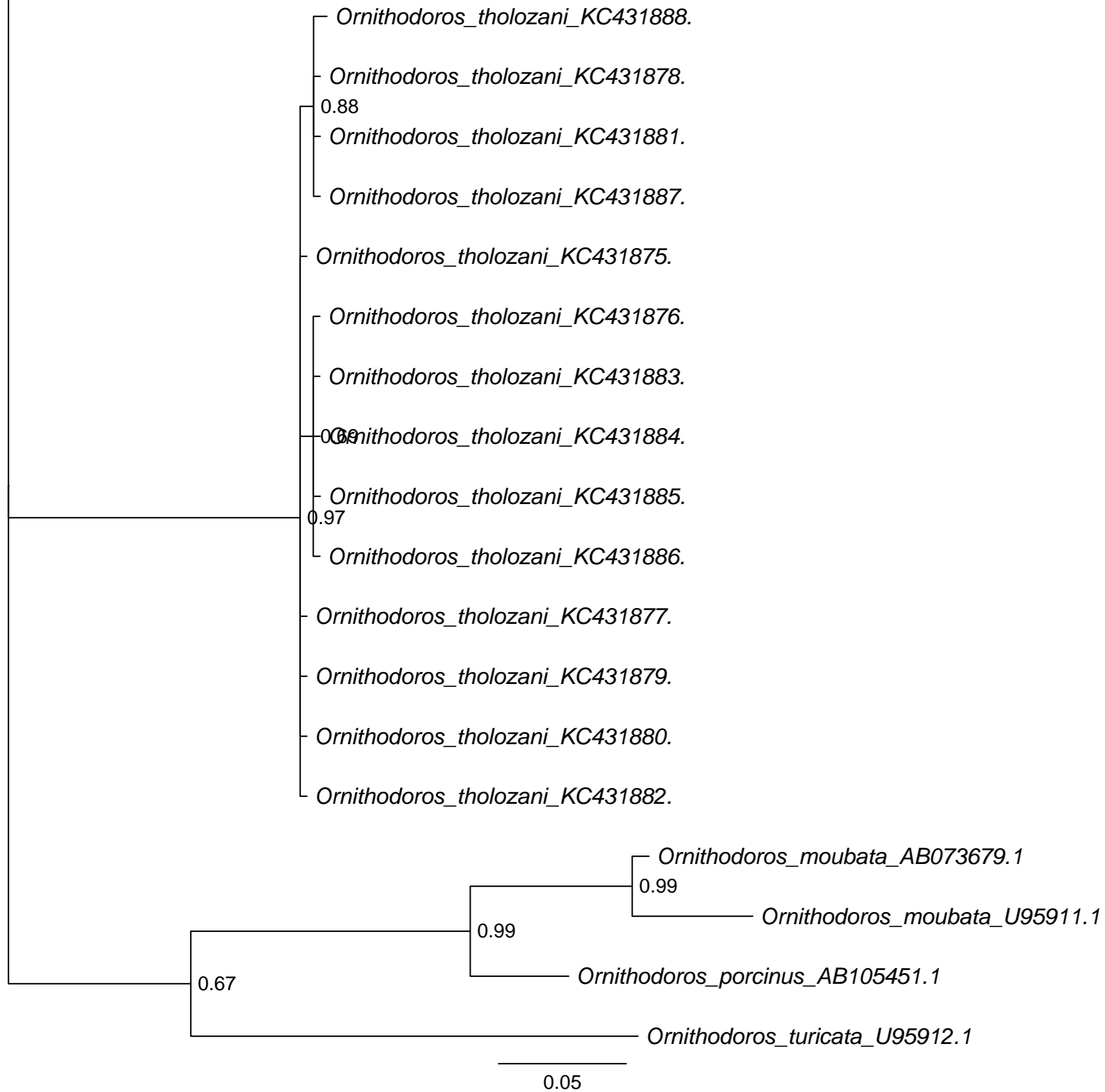

Supplement: Additional file 9 — Appendix S4. The results of Bayesian analyses. [file 1756-3305-7-93-S9.zip › Appendix.S4/12S Bayesian analyses/Ornithodoros 12S.pdf]

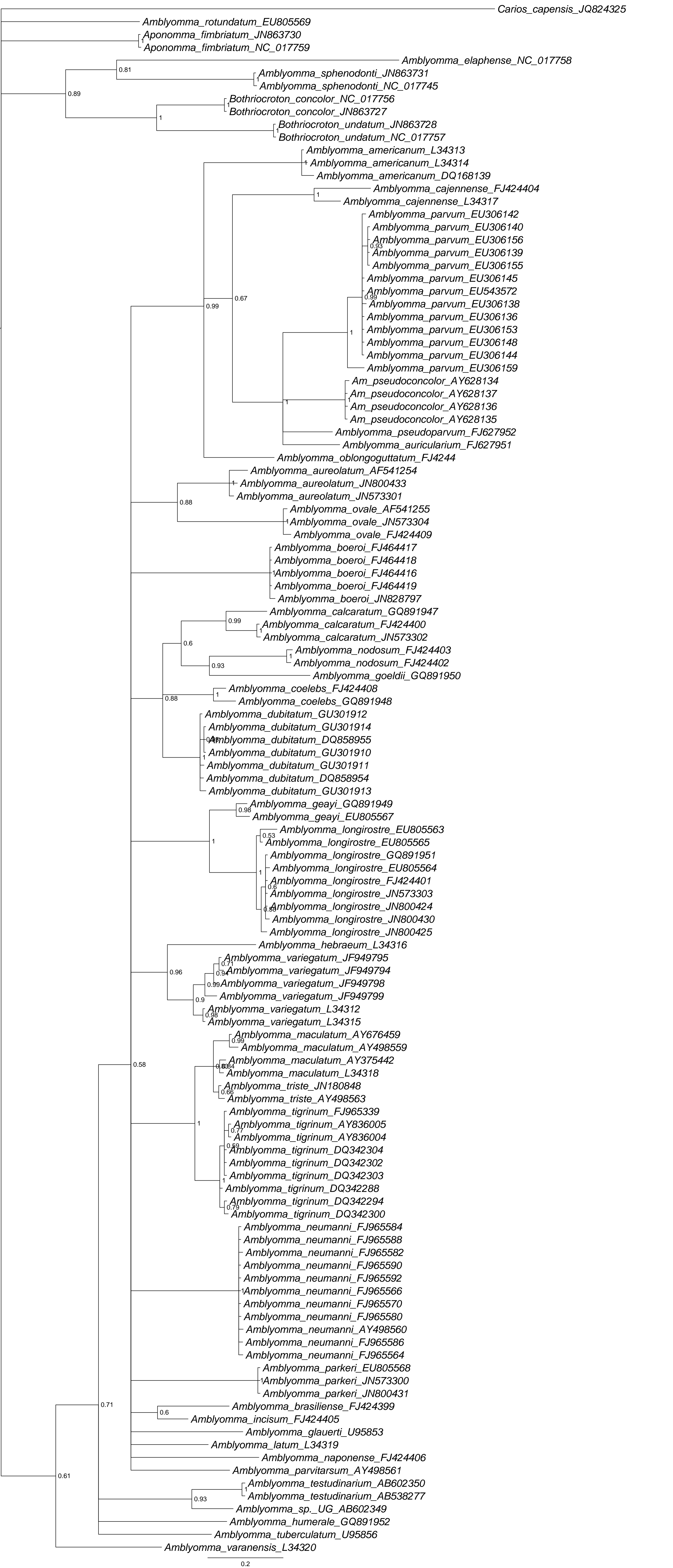

Supplement: Additional file 9 — Appendix S4. The results of Bayesian analyses. [file 1756-3305-7-93-S9.zip › Appendix.S4/16S rDNA Bayesian analyses/Amblyomma 16S.pdf]

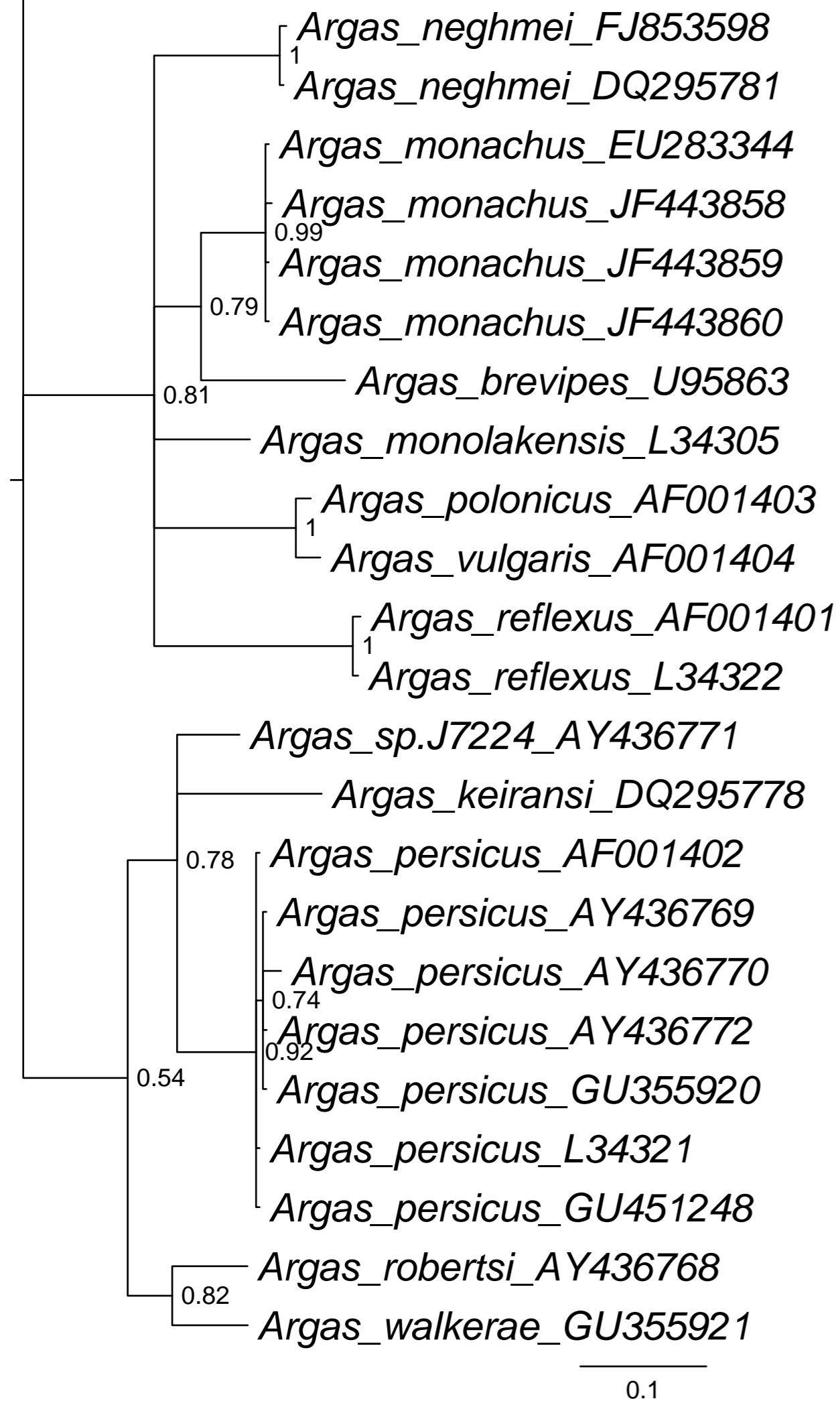

Supplement: Additional file 9 — Appendix S4. The results of Bayesian analyses. [file 1756-3305-7-93-S9.zip › Appendix.S4/16S rDNA Bayesian analyses/Argas 16S.pdf]

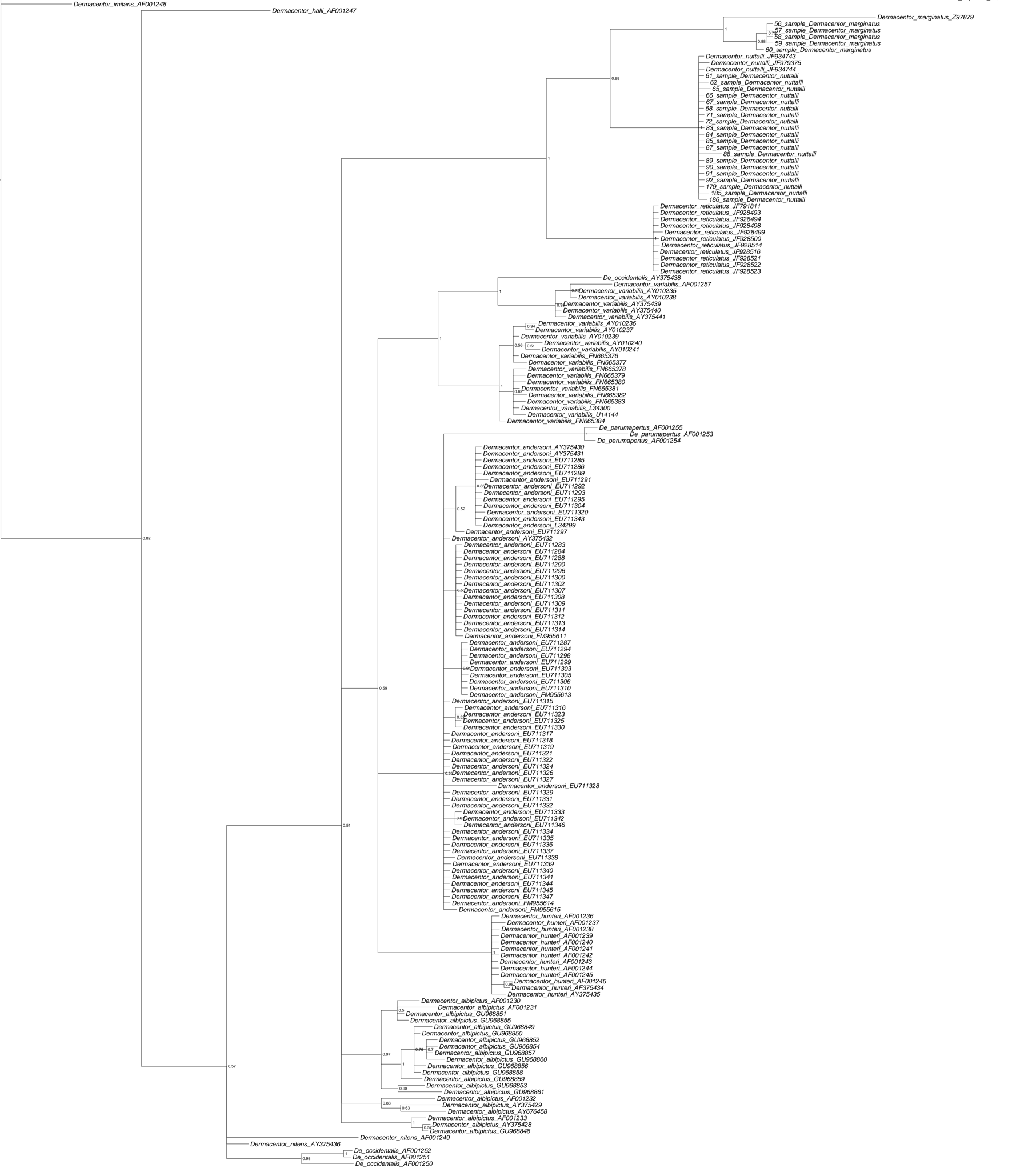

Supplement: Additional file 9 — Appendix S4. The results of Bayesian analyses. [file 1756-3305-7-93-S9.zip › Appendix.S4/16S rDNA Bayesian analyses/Dermacentor 16S.pdf]

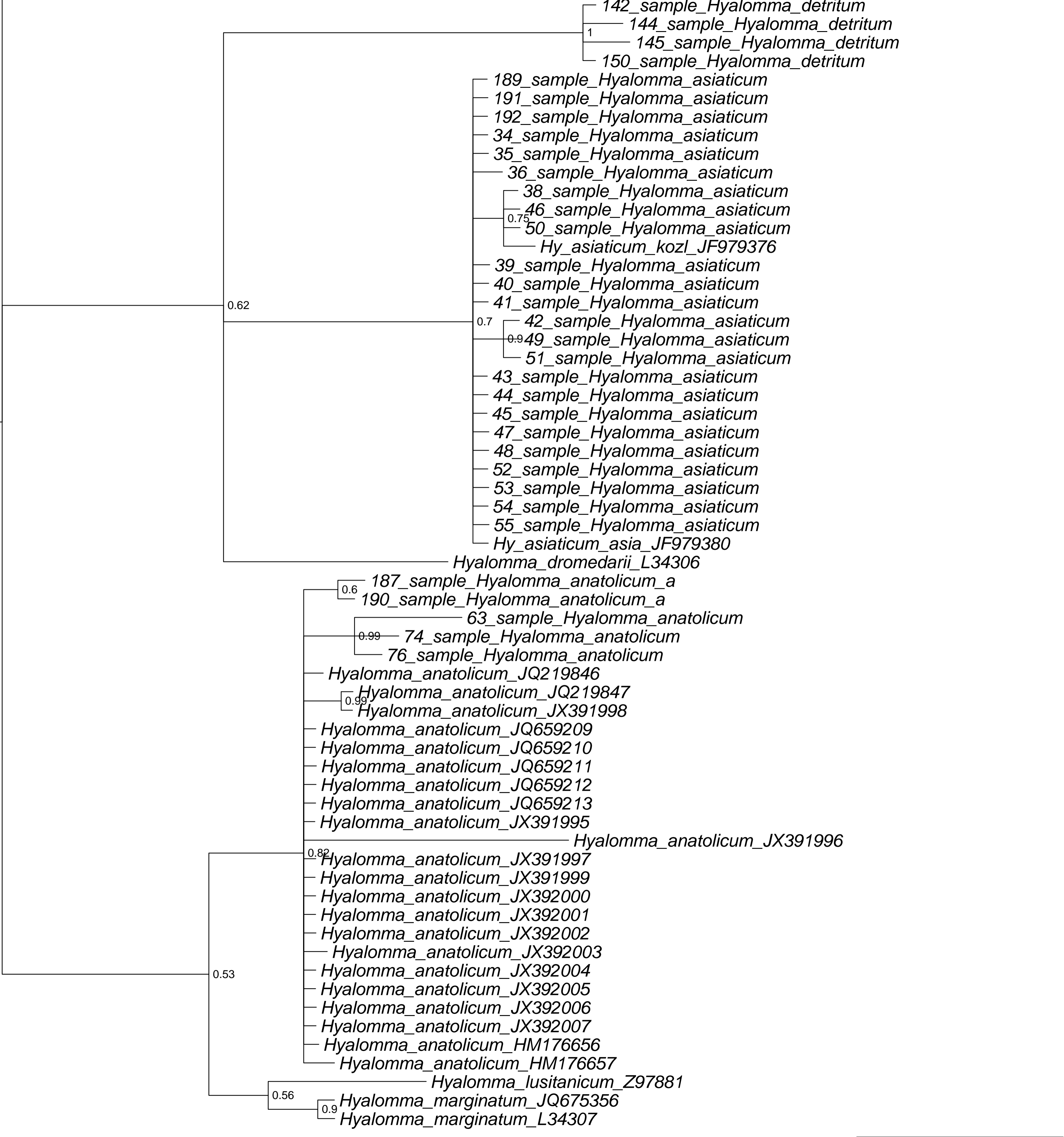

Supplement: Additional file 9 — Appendix S4. The results of Bayesian analyses. [file 1756-3305-7-93-S9.zip › Appendix.S4/16S rDNA Bayesian analyses/Hyalomma 16S.pdf]

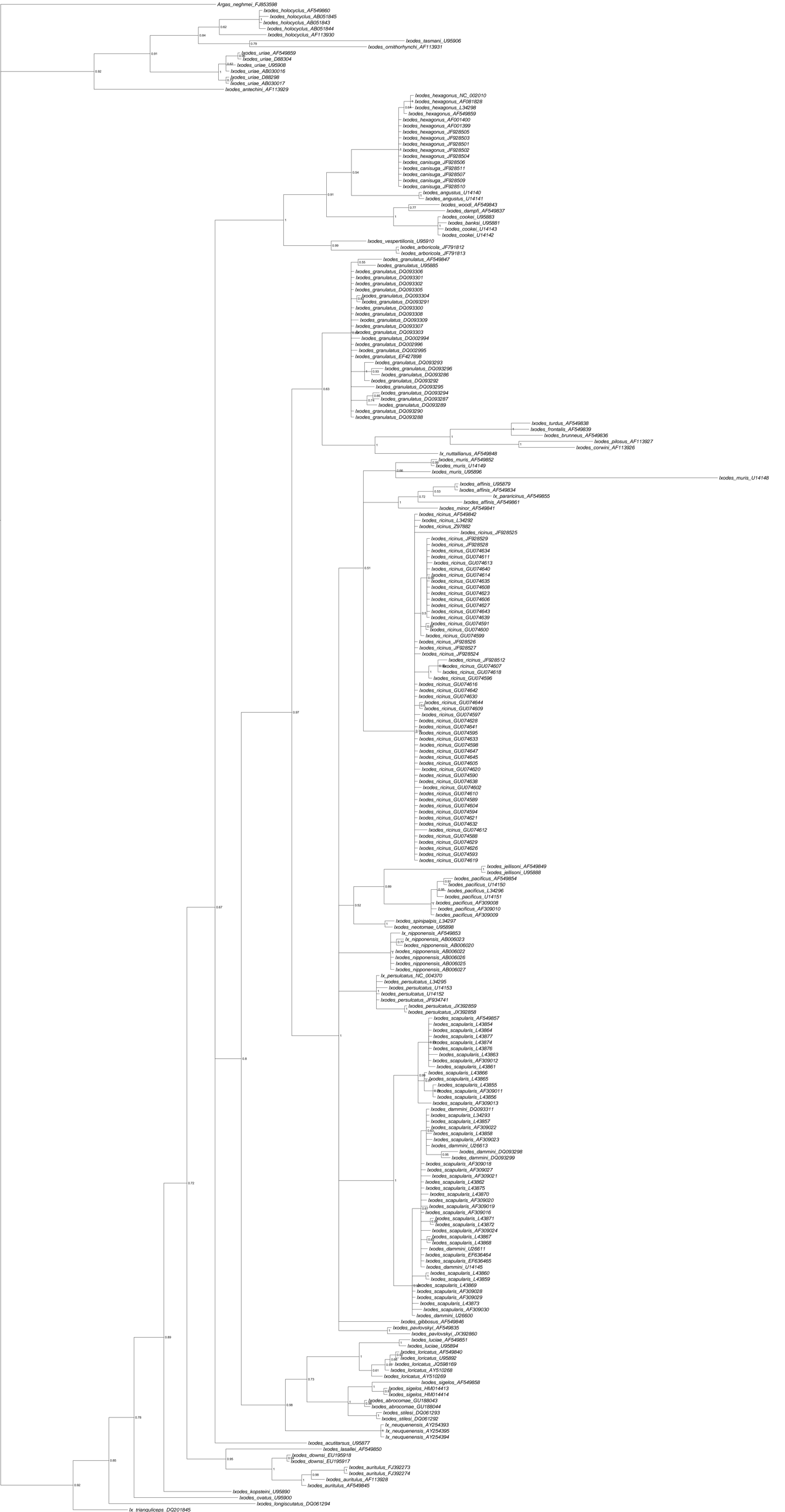

Supplement: Additional file 9 — Appendix S4. The results of Bayesian analyses. [file 1756-3305-7-93-S9.zip › Appendix.S4/16S rDNA Bayesian analyses/Ixodes 16S.pdf]

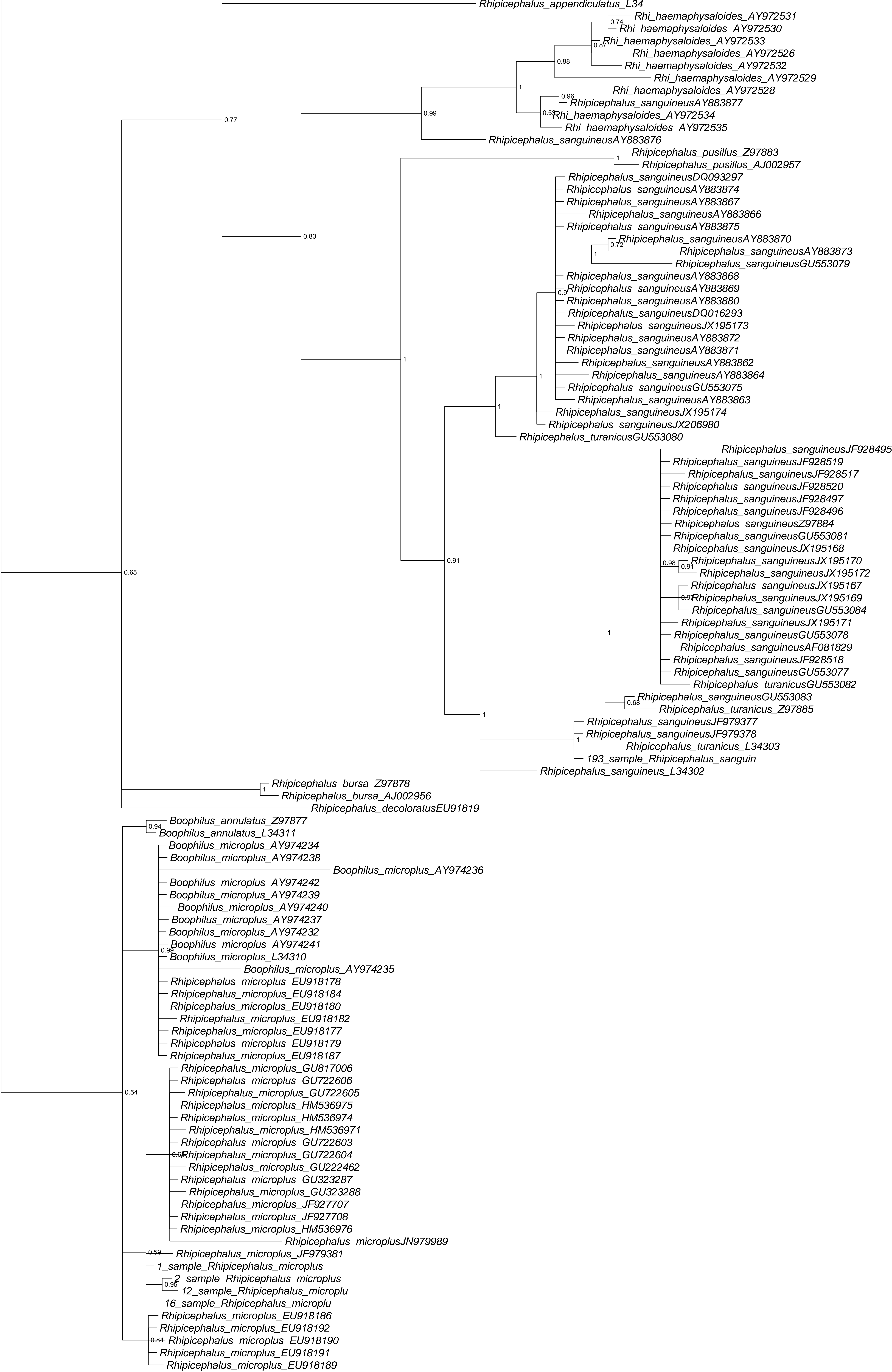

Supplement: Additional file 9 — Appendix S4. The results of Bayesian analyses. [file 1756-3305-7-93-S9.zip › Appendix.S4/16S rDNA Bayesian analyses/Rhipicephalus 16S.pdf]

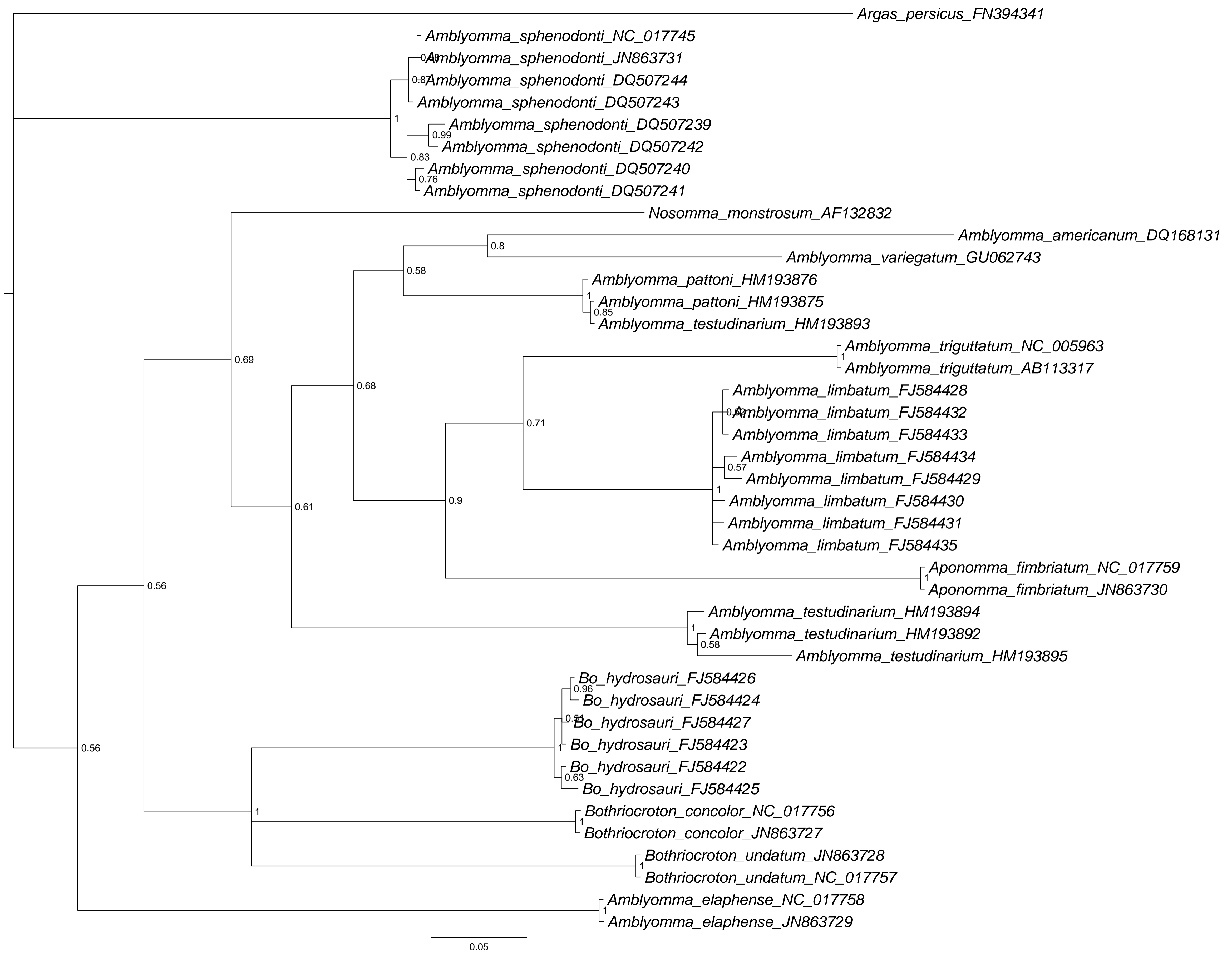

Supplement: Additional file 9 — Appendix S4. The results of Bayesian analyses. [file 1756-3305-7-93-S9.zip › Appendix.S4/COI Bayesian analyses/Amblyomma COI.pdf]

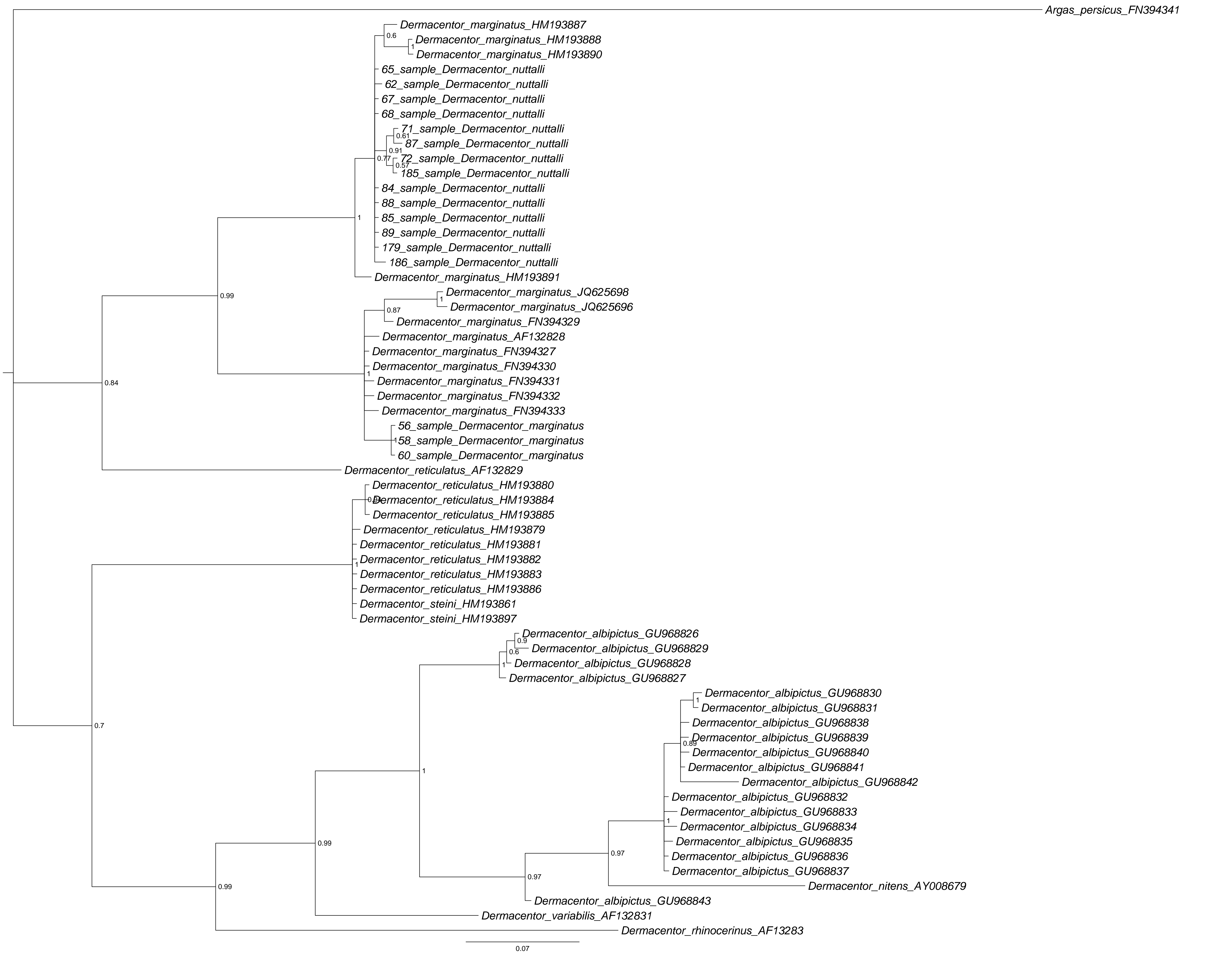

Supplement: Additional file 9 — Appendix S4. The results of Bayesian analyses. [file 1756-3305-7-93-S9.zip › Appendix.S4/COI Bayesian analyses/Dermacentor COI.pdf]

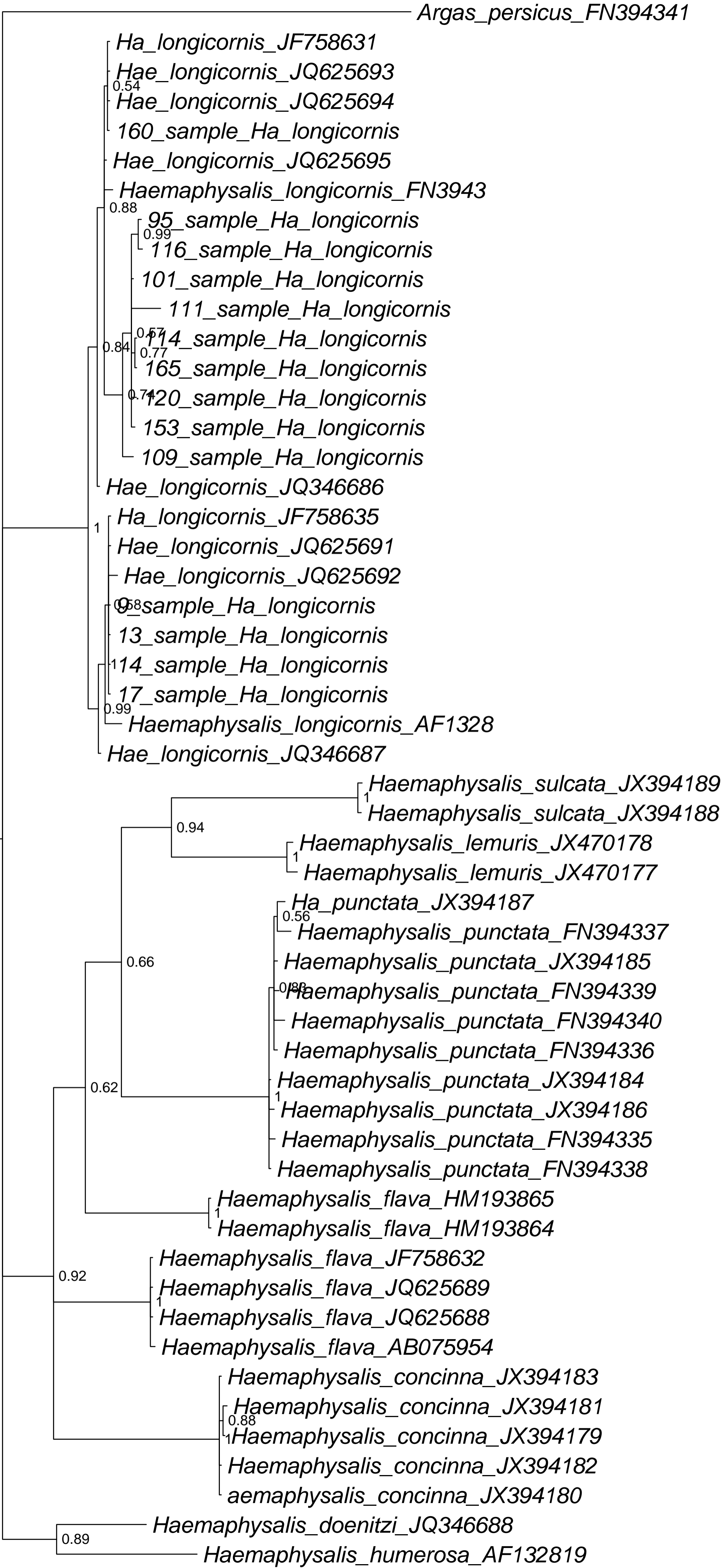

0.06

Supplement: Additional file 9 — Appendix S4. The results of Bayesian analyses. [file 1756-3305-7-93-S9.zip › Appendix.S4/COI Bayesian analyses/Haemaphysalis COI.pdf]

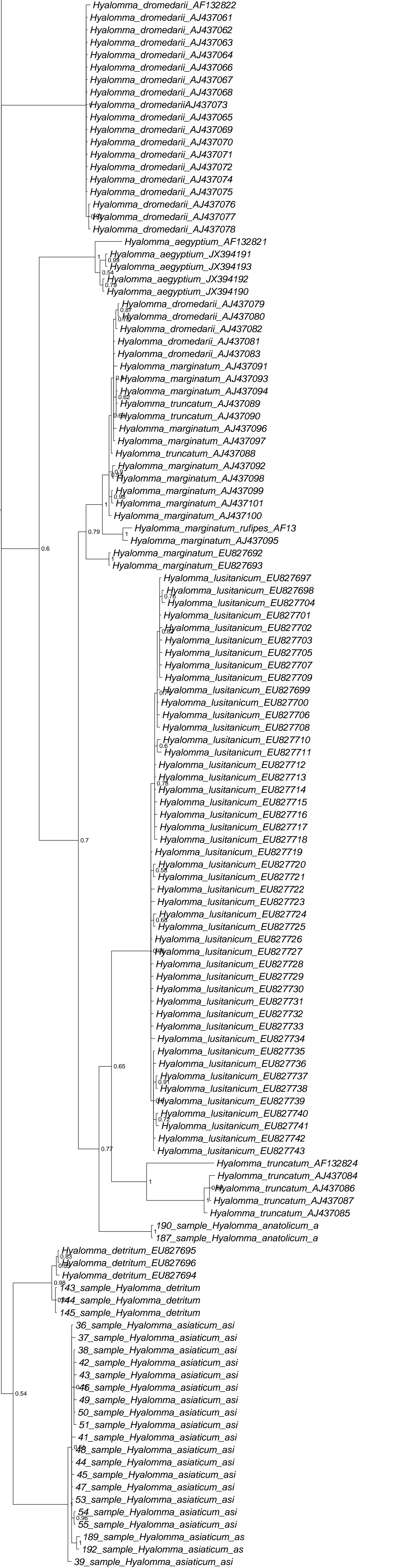

Supplement: Additional file 9 — Appendix S4. The results of Bayesian analyses. [file 1756-3305-7-93-S9.zip › Appendix.S4/COI Bayesian analyses/Hyalomma COI.pdf]

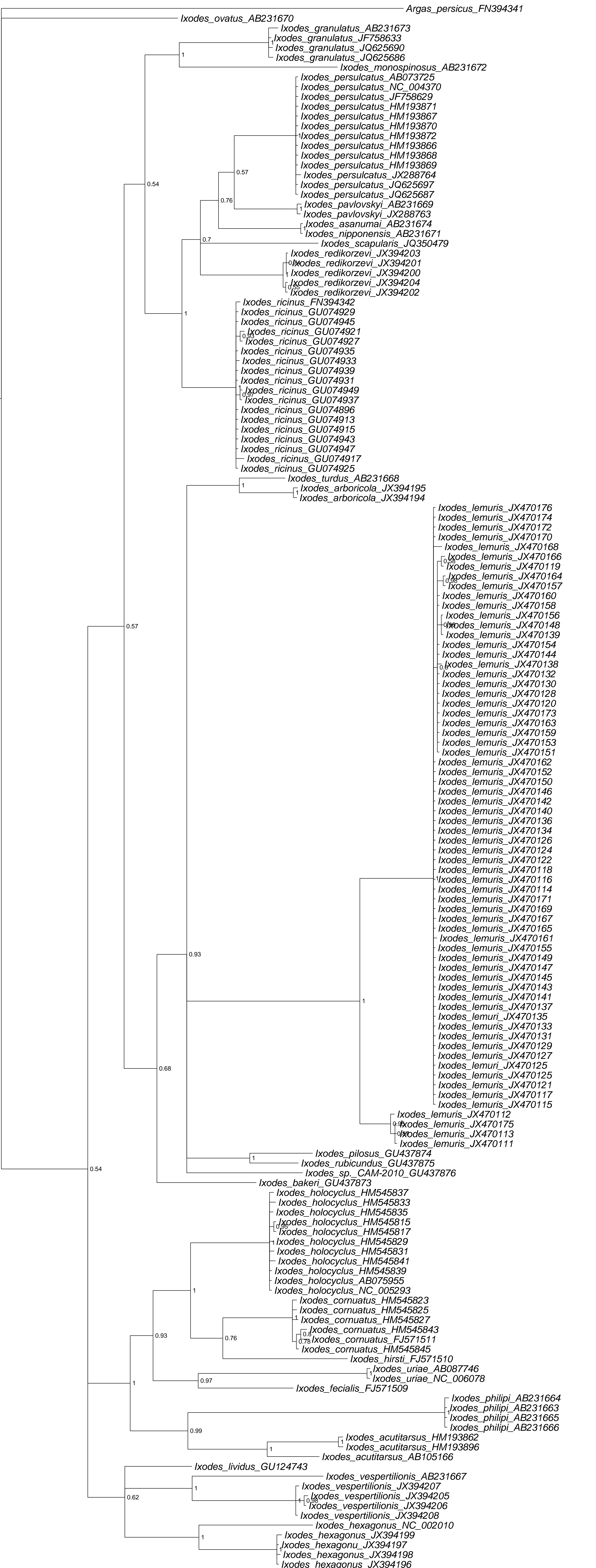

Supplement: Additional file 9 — Appendix S4. The results of Bayesian analyses. [file 1756-3305-7-93-S9.zip › Appendix.S4/COI Bayesian analyses/Ixodes COI.pdf]

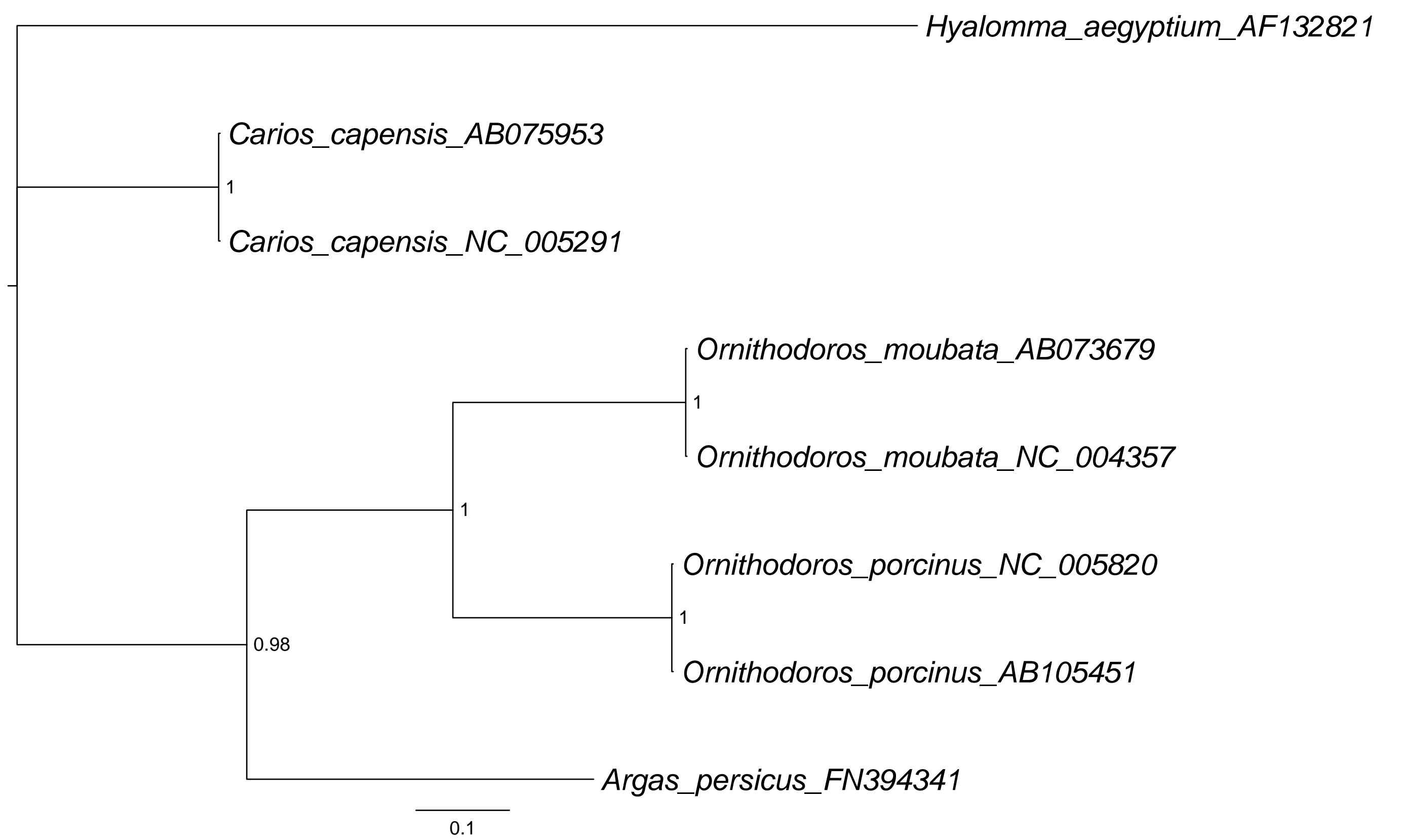

Supplement: Additional file 9 — Appendix S4. The results of Bayesian analyses. [file 1756-3305-7-93-S9.zip › Appendix.S4/COI Bayesian analyses/Ornithodoros COI.pdf]

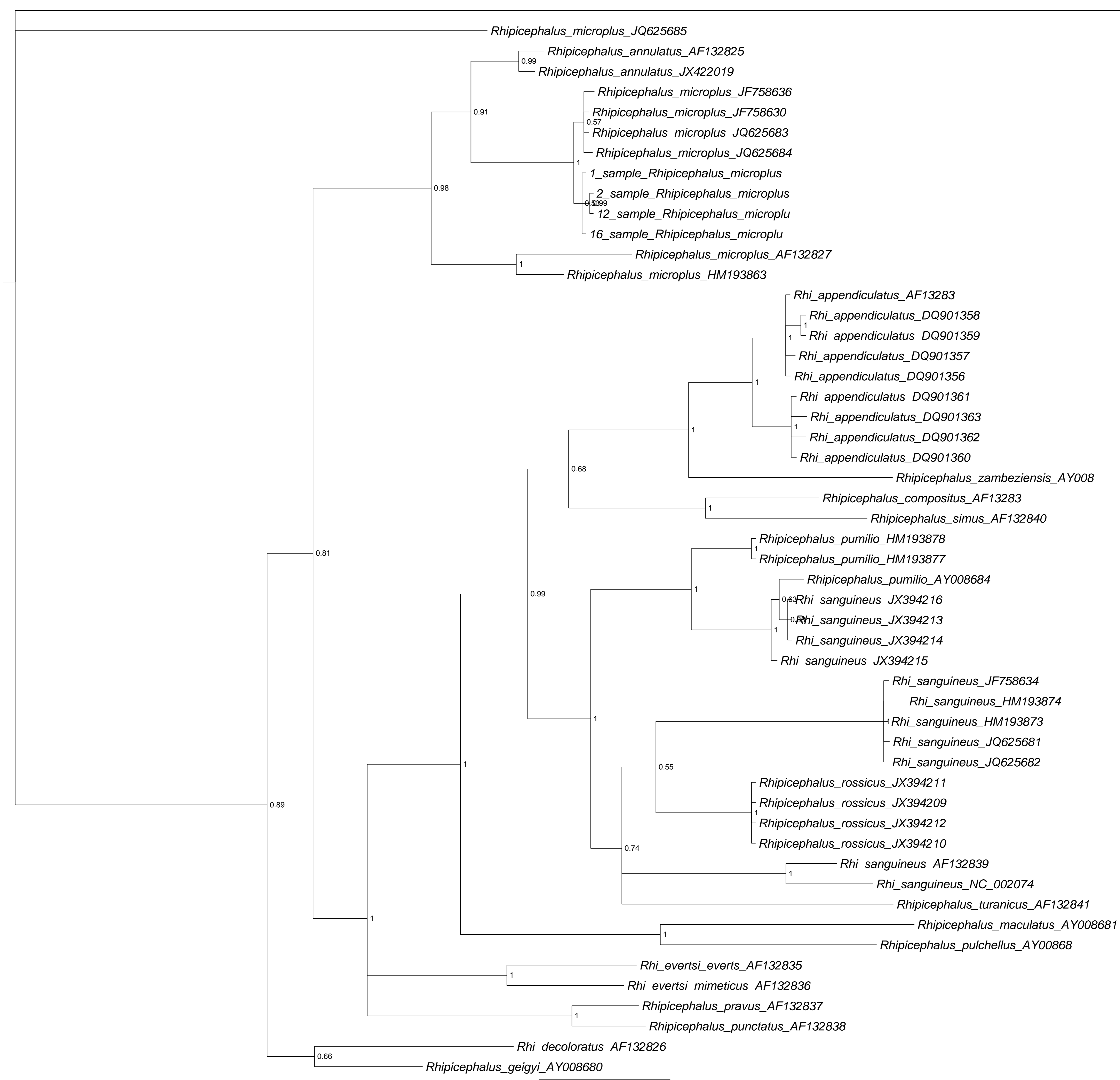

Supplement: Additional file 9 — Appendix S4. The results of Bayesian analyses. [file 1756-3305-7-93-S9.zip › Appendix.S4/COI Bayesian analyses/Rhipicephalus COI.pdf]

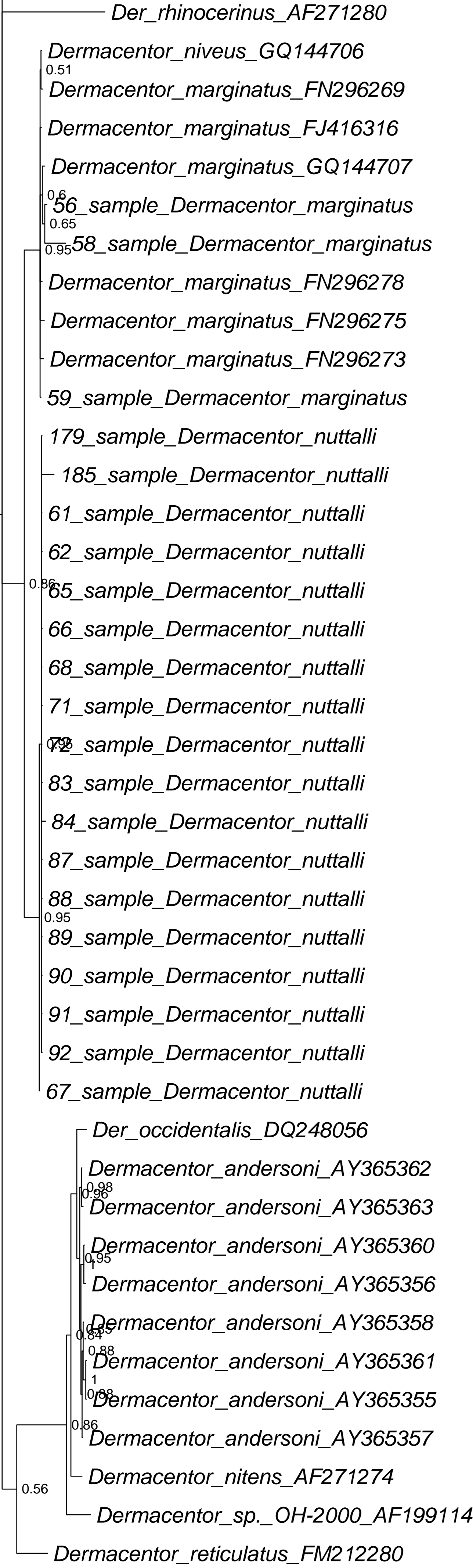

Supplement: Additional file 9 — Appendix S4. The results of Bayesian analyses. [file 1756-3305-7-93-S9.zip › Appendix.S4/ITS2 Bayesian analyses/Dermacentor ITS2.pdf]

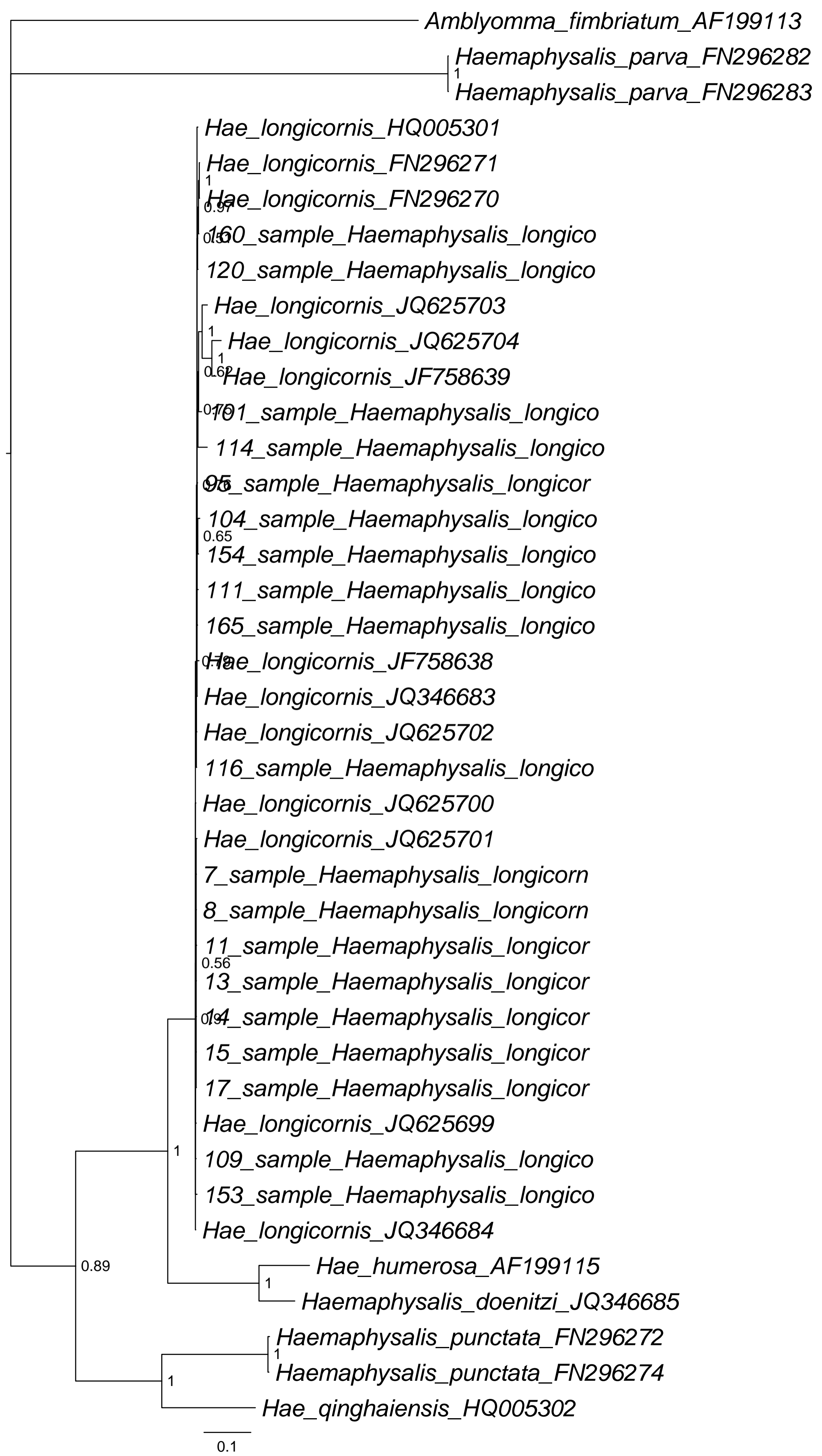

Supplement: Additional file 9 — Appendix S4. The results of Bayesian analyses. [file 1756-3305-7-93-S9.zip › Appendix.S4/ITS2 Bayesian analyses/Haemaphysalis ITS2.pdf]

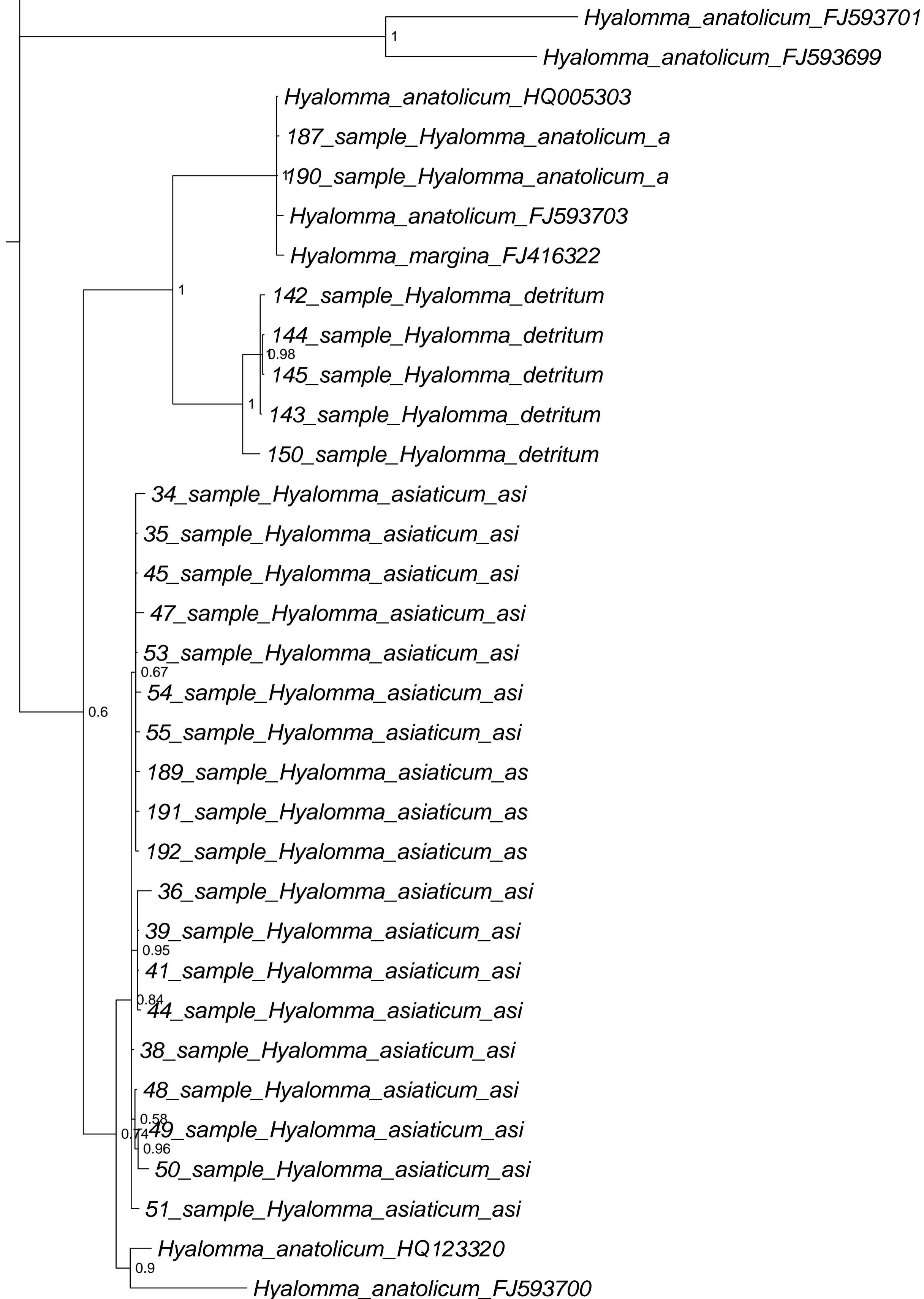

Supplement: Additional file 9 — Appendix S4. The results of Bayesian analyses. [file 1756-3305-7-93-S9.zip › Appendix.S4/ITS2 Bayesian analyses/Hyalomma ITS2.pdf]

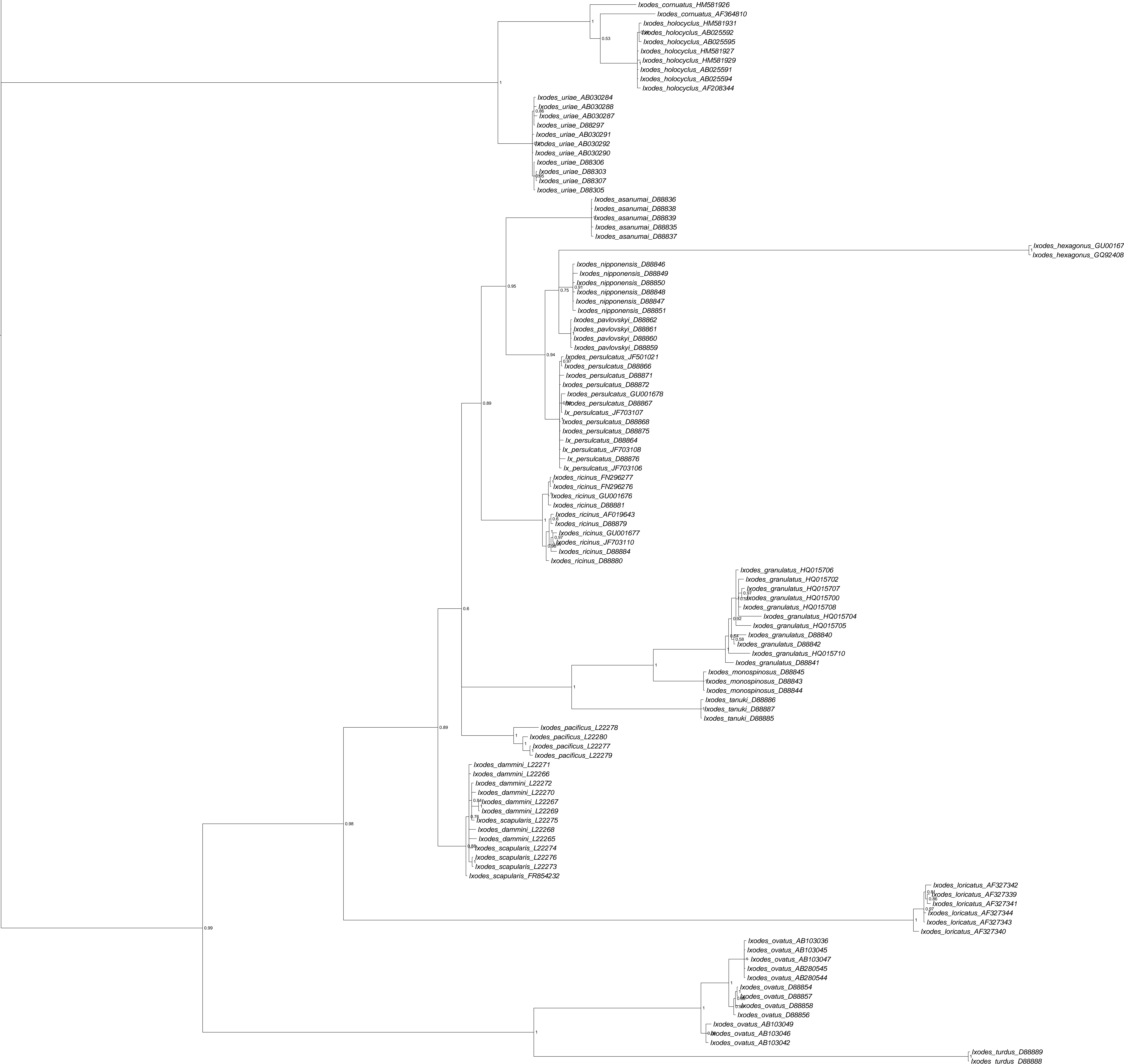

Supplement: Additional file 9 — Appendix S4. The results of Bayesian analyses. [file 1756-3305-7-93-S9.zip › Appendix.S4/ITS2 Bayesian analyses/Ixodes ITS2.pdf]

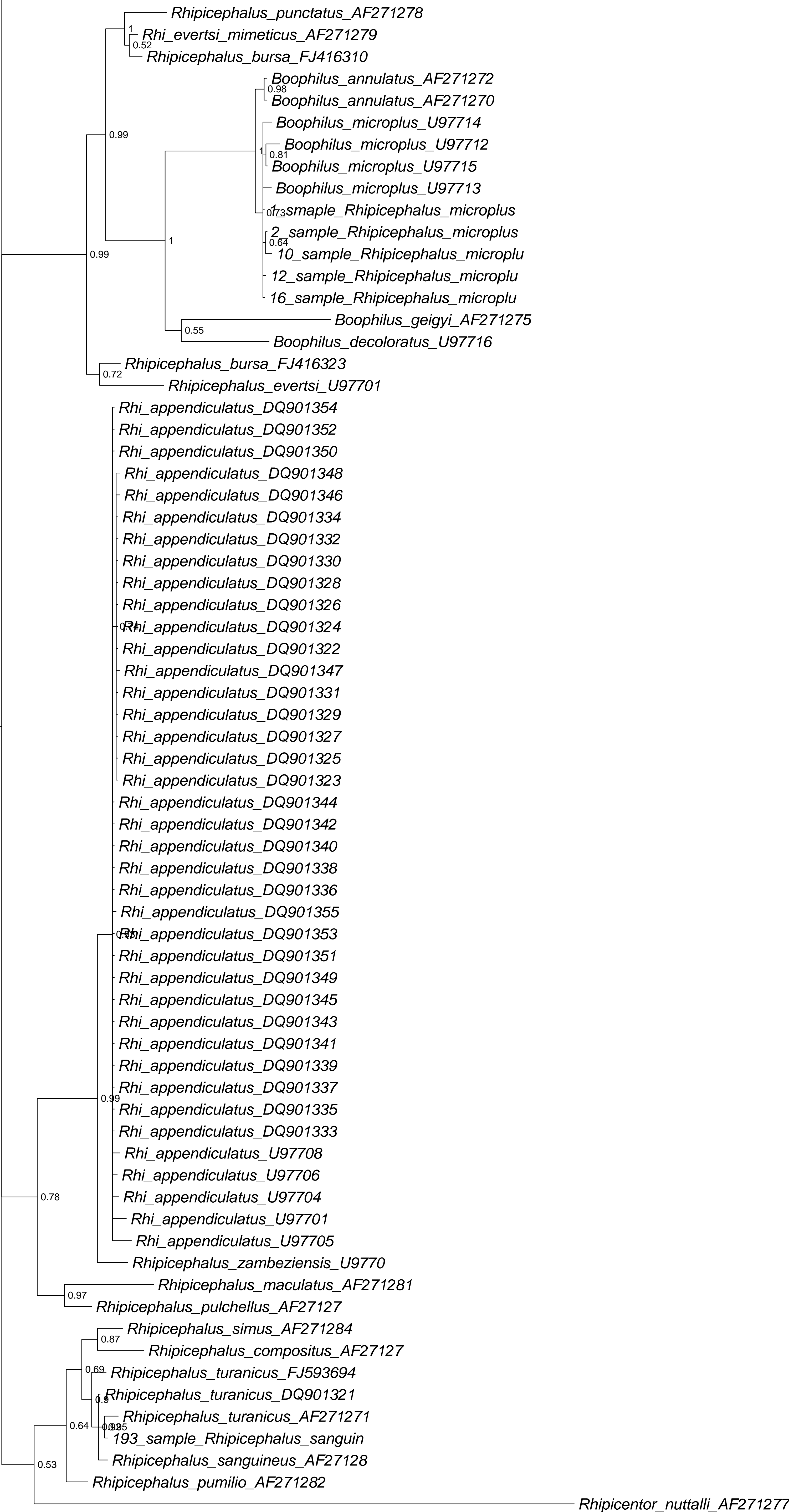

Supplement: Additional file 9 — Appendix S4. The results of Bayesian analyses. [file 1756-3305-7-93-S9.zip › Appendix.S4/ITS2 Bayesian analyses/Rhipicephalus ITS2.pdf]

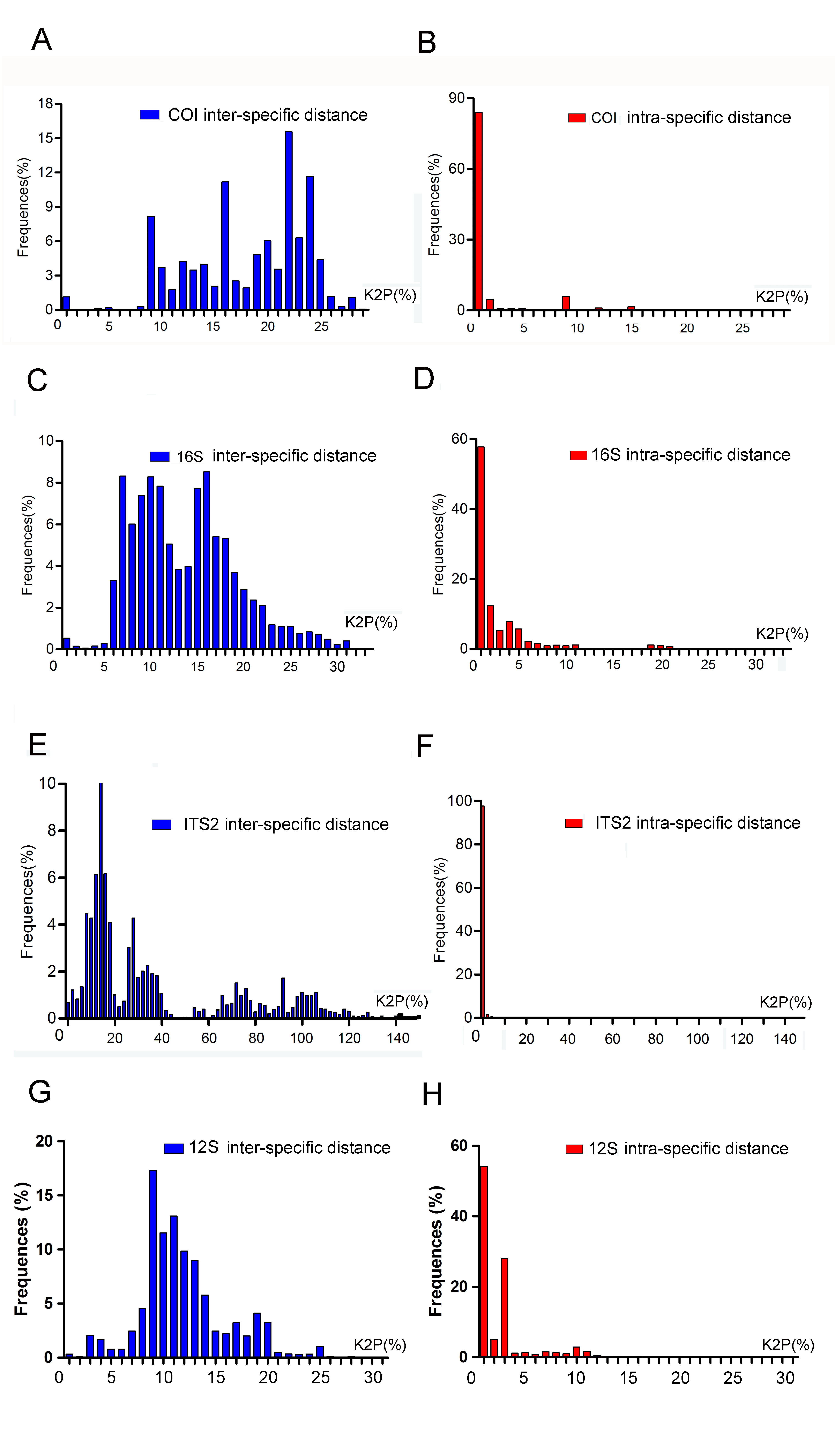

Supplement: Additional file 10: Figure S1 — Frequency distributions of K2P for COI, 16S rDNA, ITS2 and 12S rDNA within and among species based on data set 3. [file 1756-3305-7-93-S10.tiff]

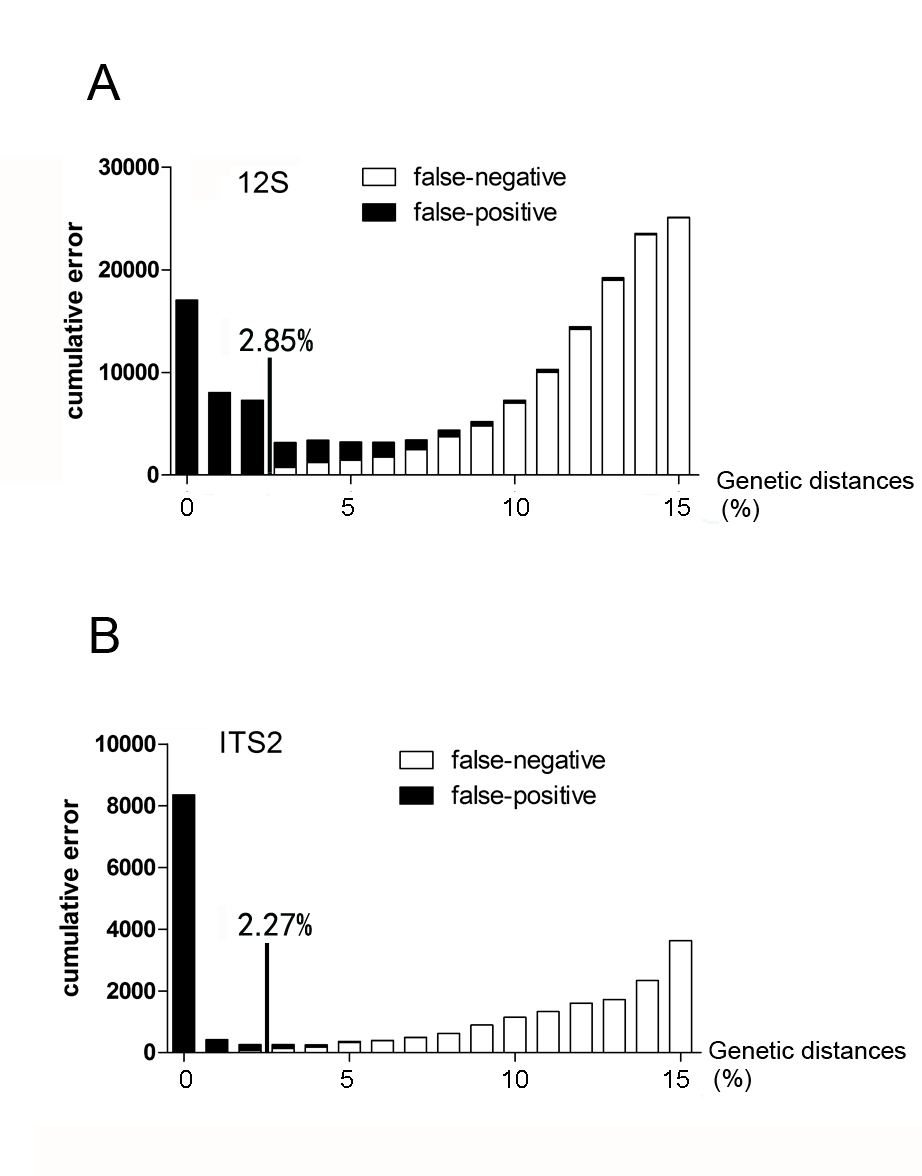

Supplement: Additional file 11: Figure S2 — Cumulative error distributions among the species of ticks calculated from (A) 12S and (B) ITS2. [file 1756-3305-7-93-S11.tiff]
