# Supplementary material for: Assessment of four DNA fragments (COI, 16S rDNA, ITS2, 12S rDNA) for species identification of the Ixodida (Acari: Ixodida)
Source: Parasit Vectors. 2014 Mar 3;7:93. doi: 10.1186/1756-3305-7-93 (PMC3945964; doi:10.1186/1756-3305-7-93)
Supplement: Additional file 9 — Appendix S4. The results of Bayesian analyses. [file Haemaphysalis 12S.pdf › Appendix.S4/12S Bayesian analyses/Haemaphysalis 12S.pdf]

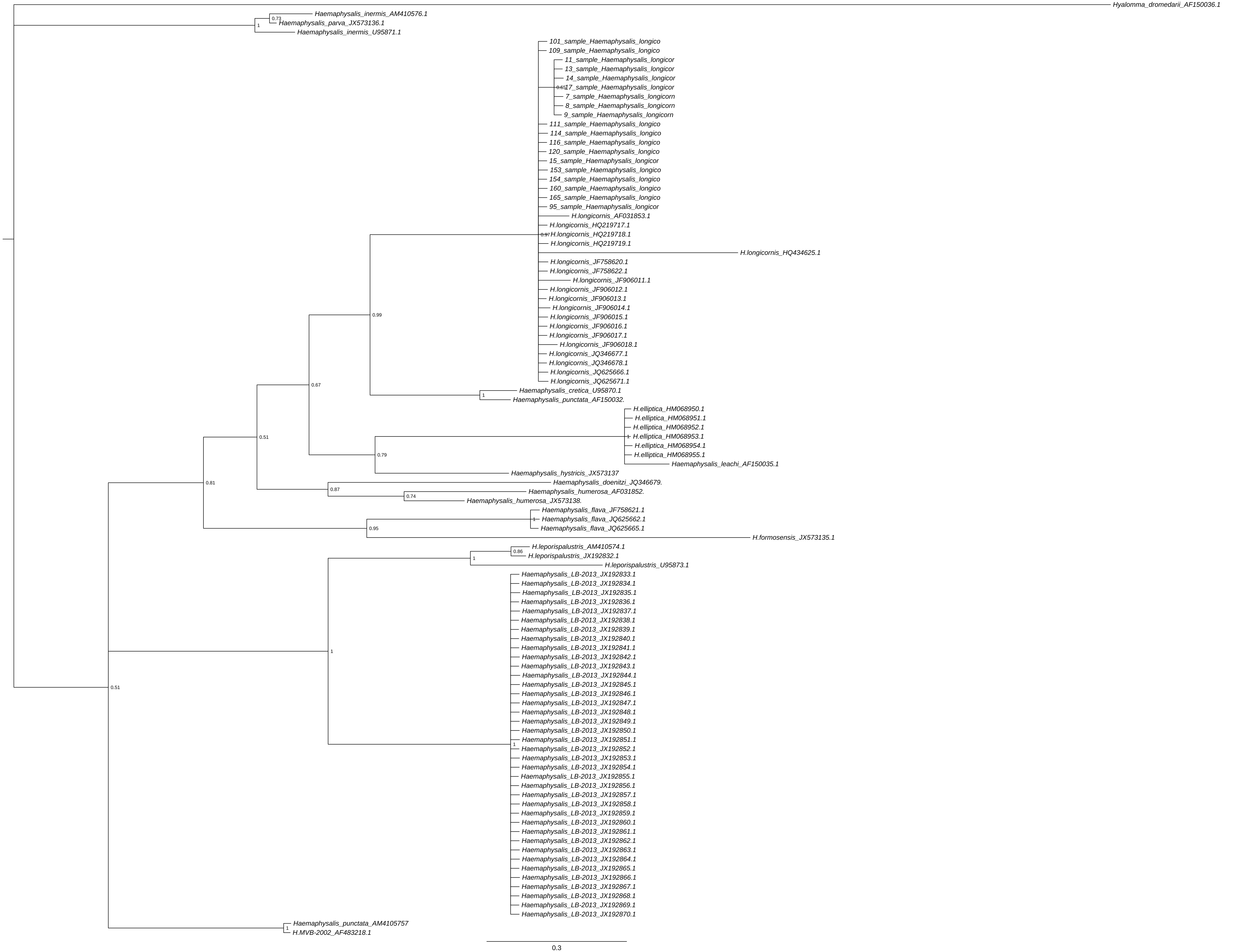
Hyalomma_dromedarii_AF150036.1
Haemaphysalis_inermis_AM410576.1
Haemaphysalis_parva_JX573136.1
Haemaphysalis_inermis_U95871.1
101_sample_Haemaphysalis_longico
109_sample_Haemaphysalis_longico
11_sample_Haemaphysalis_longicor
13_sample_Haemaphysalis_longicor
14_sample_Haemaphysalis_longicor
17_sample_Haemaphysalis_longicor
7_sample_Haemaphysalis_longicorn
8_sample_Haemaphysalis_longicorn
9_sample_Haemaphysalis_longicorn
111_sample_Haemaphysalis_longico
114_sample_Haemaphysalis_longico
116_sample_Haemaphysalis_longico
120_sample_Haemaphysalis_longico
15_sample_Haemaphysalis_longicor
153_sample_Haemaphysalis_longico
154_sample_Haemaphysalis_longico
160_sample_Haemaphysalis_longico
165_sample_Haemaphysalis_longico
95_sample_Haemaphysalis_longicor
H.longicornis_AF031853.1
H.longicornis_HQ219717.1
H.longicornis_HQ219718.1
H.longicornis_HQ219719.1
H.longicornis_HQ434625.1
H.longicornis_JF758620.1
H.longicornis_JF758622.1
H.longicornis_JF906011.1
H.longicornis_JF906012.1
H.longicornis_JF906013.1
H.longicornis_JF906014.1
H.longicornis_JF906015.1
H.longicornis_JF906016.1
H.longicornis_JF906017.1
H.longicornis_JF906018.1
H.longicornis_JQ346677.1
H.longicornis_JQ346678.1
H.longicornis_JQ625666.1
H.longicornis_JQ625671.1
Haemaphysalis_cretica_U95870.1
Haemaphysalis_punctata_AF150032.
H.elliptica_HM068950.1
H.elliptica_HM068951.1
H.elliptica_HM068952.1
H.elliptica_HM068953.1
H.elliptica_HM068954.1
H.elliptica_HM068955.1
Haemaphysalis_leachi_AF150035.1
Haemaphysalis_hystricis_JX573137
Haemaphysalis_doenitzi_JQ346679.
Haemaphysalis_humerosa_AF031852.
Haemaphysalis_humerosa_JX573138.
Haemaphysalis_flava_JF758621.1
Haemaphysalis_flava_JQ625662.1
Haemaphysalis_flava_JQ625665.1
H.formosensis_JX573135.1
H.leporispalustris_AM410574.1
H.leporispalustris_JX192832.1
H.leporispalustris_U95873.1
Haemaphysalis_LB-2013_JX192833.1
Haemaphysalis_LB-2013_JX192834.1
Haemaphysalis_LB-2013_JX192835.1
Haemaphysalis_LB-2013_JX192836.1
Haemaphysalis_LB-2013_JX192837.1
Haemaphysalis_LB-2013_JX192838.1
Haemaphysalis_LB-2013_JX192839.1
Haemaphysalis_LB-2013_JX192840.1
Haemaphysalis_LB-2013_JX192841.1
Haemaphysalis_LB-2013_JX192842.1
Haemaphysalis_LB-2013_JX192843.1
Haemaphysalis_LB-2013_JX192844.1
Haemaphysalis_LB-2013_JX192845.1
Haemaphysalis_LB-2013_JX192846.1
Haemaphysalis_LB-2013_JX192847.1
Haemaphysalis_LB-2013_JX192848.1
Haemaphysalis_LB-2013_JX192849.1
Haemaphysalis_LB-2013_JX192850.1
Haemaphysalis_LB-2013_JX192851.1
Haemaphysalis_LB-2013_JX192852.1
Haemaphysalis_LB-2013_JX192853.1
Haemaphysalis_LB-2013_JX192854.1
Haemaphysalis_LB-2013_JX192855.1
Haemaphysalis_LB-2013_JX192856.1
Haemaphysalis_LB-2013_JX192857.1
Haemaphysalis_LB-2013_JX192858.1
Haemaphysalis_LB-2013_JX192859.1
Haemaphysalis_LB-2013_JX192860.1
Haemaphysalis_LB-2013_JX192861.1
Haemaphysalis_LB-2013_JX192862.1
Haemaphysalis_LB-2013_JX192863.1
Haemaphysalis_LB-2013_JX192864.1
Haemaphysalis_LB-2013_JX192865.1
Haemaphysalis_LB-2013_JX192866.1
Haemaphysalis_LB-2013_JX192867.1
Haemaphysalis_LB-2013_JX192868.1
Haemaphysalis_LB-2013_JX192869.1
Haemaphysalis_LB-2013_JX192870.1
Haemaphysalis_punctata_AM4105757
H.MVB-2002_AF483218.1
0.3
