# Supplementary material for: Assessment of four DNA fragments (COI, 16S rDNA, ITS2, 12S rDNA) for species identification of the Ixodida (Acari: Ixodida)
Source: Parasit Vectors. 2014 Mar 3;7:93. doi: 10.1186/1756-3305-7-93 (PMC3945964; doi:10.1186/1756-3305-7-93)

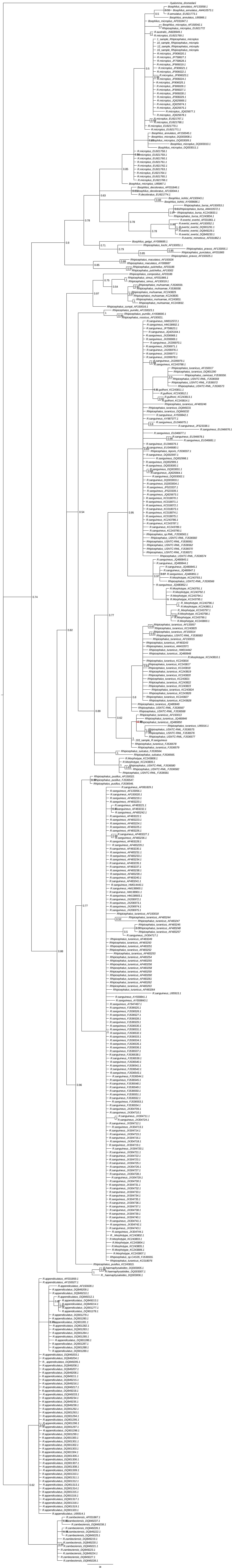

Supplement: Additional file 9 — Appendix S4. The results of Bayesian analyses. [file Rhipicephalus 12S.pdf › Appendix.S4/12S Bayesian analyses/Rhipicephalus 12S.pdf]
