# Supplementary material for: Assessment of four DNA fragments (COI, 16S rDNA, ITS2, 12S rDNA) for species identification of the Ixodida (Acari: Ixodida)
Source: Parasit Vectors. 2014 Mar 3;7:93. doi: 10.1186/1756-3305-7-93 (PMC3945964; doi:10.1186/1756-3305-7-93)
Supplement: Additional file 9 — Appendix S4. The results of Bayesian analyses. [file Ornithodoros 16S.pdf › Appendix.S4/16S rDNA Bayesian analyses/Ornithodoros 16S.pdf]

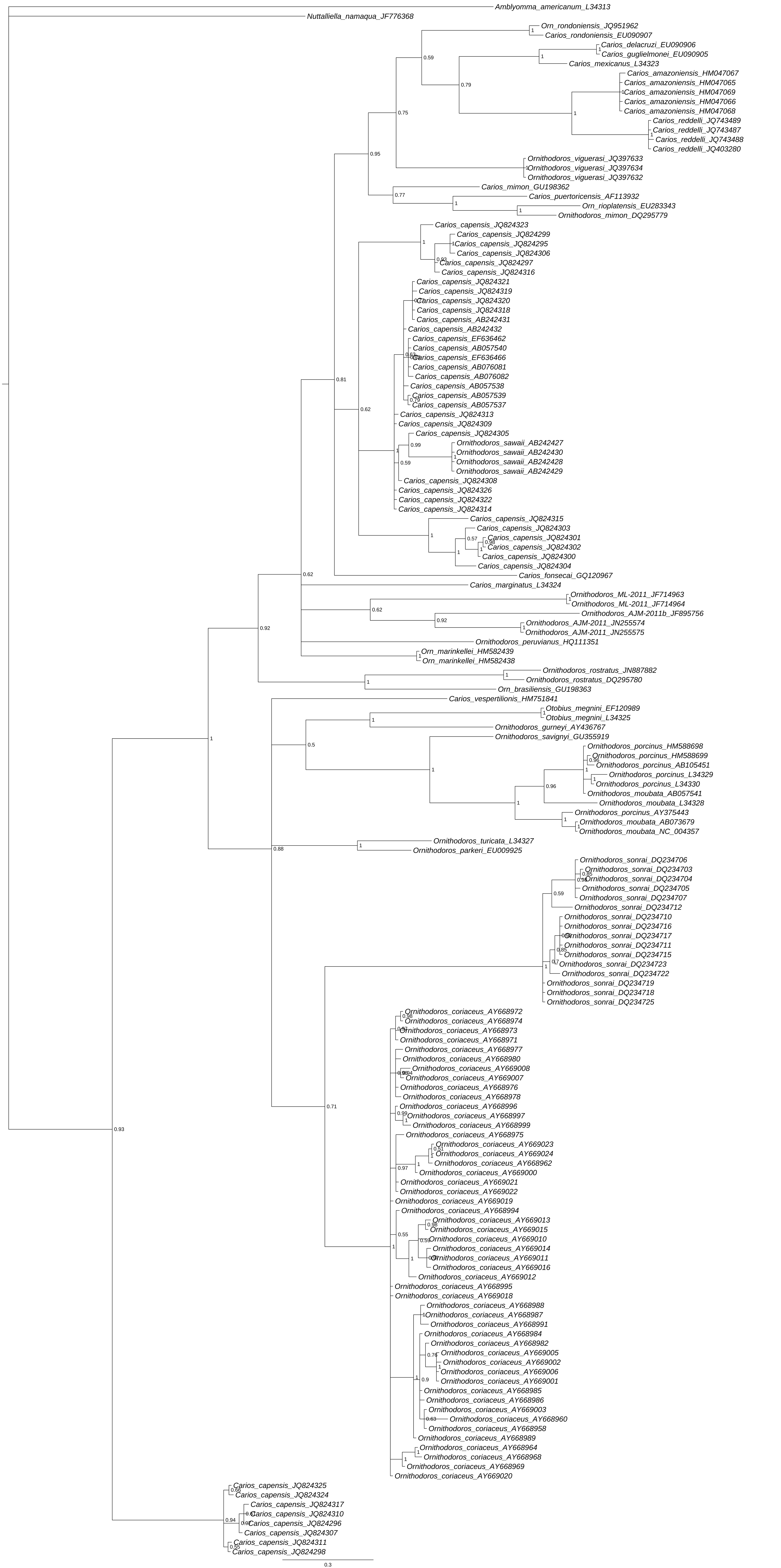
Amblyomma_americanum_L34313
Nuttalliella_namaqua_JF776368
Orn_rondoniensis_JQ951962
Carios_rondoniensis_EU090907
Carios_delacruzi_EU090906
Carios_guglielmonei_EU090905
Carios_mexicanus_L34323
Carios_amazoniensis_HM047067
Carios_amazoniensis_HM047065
Carios_amazoniensis_HM047069
Carios_amazoniensis_HM047066
Carios_amazoniensis_HM047068
Carios_reddelli_JQ743489
Carios_reddelli_JQ743487
Carios_reddelli_JQ743488
Carios_reddelli_JQ403280
Ornithodoros_viguerasi_JQ397633
Ornithodoros_viguerasi_JQ397634
Ornithodoros_viguerasi_JQ397632
Carios_mimon_GU198362
Carios_puertoricensis_AF113932
Orn_rioplatensis_EU283343
Ornithodoros_mimon_DQ295779
Carios_capensis_JQ824323
Carios_capensis_JQ824299
Carios_capensis_JQ824295
Carios_capensis_JQ824306
Carios_capensis_JQ824297
Carios_capensis_JQ824316
Carios_capensis_JQ824321
Carios_capensis_JQ824319
Carios_capensis_JQ824320
Carios_capensis_JQ824318
Carios_capensis_AB242431
Carios_capensis_AB242432
Carios_capensis_EF636462
Carios_capensis_AB057540
Carios_capensis_EF636466
Carios_capensis_AB076081
Carios_capensis_AB076082
Carios_capensis_AB057538
Carios_capensis_AB057539
Carios_capensis_AB057537
Carios_capensis_JQ824313
Carios_capensis_JQ824309
Carios_capensis_JQ824305
Ornithodoros_sawaii_AB242427
Ornithodoros_sawaii_AB242430
Ornithodoros_sawaii_AB242428
Ornithodoros_sawaii_AB242429
Carios_capensis_JQ824308
Carios_capensis_JQ824326
Carios_capensis_JQ824322
Carios_capensis_JQ824314
Carios_capensis_JQ824315
Carios_capensis_JQ824303
Carios_capensis_JQ824301
Carios_capensis_JQ824302
Carios_capensis_JQ824300
Carios_capensis_JQ824304
Carios_fonsecai_GQ120967
Carios_marginatus_L34324
Ornithodoros_ML-2011_JF714963
Ornithodoros_ML-2011_JF714964
Ornithodoros_AJM-2011b_JF895756
Ornithodoros_AJM-2011_JN255574
Ornithodoros_AJM-2011_JN255575
Ornithodoros_peruvianus_HQ111351
Orn_marinkellei_HM582439
Orn_marinkellei_HM582438
Ornithodoros_rostratus_JN887882
Ornithodoros_rostratus_DQ295780
Orn_brasiliensis_GU198363
Carios_vespertilionis_HM751841
Otobius_megnini_EF120989
Otobius_megnini_L34325
Ornithodoros_gurneyi_AY436767
Ornithodoros_savignyi_GU355919
Ornithodoros_porcinus_HM588698
Ornithodoros_porcinus_HM588699
Ornithodoros_porcinus_AB105451
Ornithodoros_porcinus_L34329
Ornithodoros_porcinus_L34330
Ornithodoros_moubata_AB057541
Ornithodoros_moubata_L34328
Ornithodoros_porcinus_AY375443
Ornithodoros_moubata_AB073679
Ornithodoros_moubata_NC_004357
Ornithodoros_turicata_L34327
Ornithodoros_parkeri_EU009925
Ornithodoros_sonrai_DQ234706
Ornithodoros_sonrai_DQ234703
Ornithodoros_sonrai_DQ234704
Ornithodoros_sonrai_DQ234705
Ornithodoros_sonrai_DQ234707
Ornithodoros_sonrai_DQ234712
Ornithodoros_sonrai_DQ234710
Ornithodoros_sonrai_DQ234716
Ornithodoros_sonrai_DQ234717
Ornithodoros_sonrai_DQ234711
Ornithodoros_sonrai_DQ234715
Ornithodoros_sonrai_DQ234723
Ornithodoros_sonrai_DQ234722
Ornithodoros_sonrai_DQ234719
Ornithodoros_sonrai_DQ234718
Ornithodoros_sonrai_DQ234725
Ornithodoros_coriaceus_AY668972
Ornithodoros_coriaceus_AY668974
Ornithodoros_coriaceus_AY668973
Ornithodoros_coriaceus_AY668971
Ornithodoros_coriaceus_AY668977
Ornithodoros_coriaceus_AY668980
Ornithodoros_coriaceus_AY669008
Ornithodoros_coriaceus_AY669007
Ornithodoros_coriaceus_AY668976
Ornithodoros_coriaceus_AY668978
Ornithodoros_coriaceus_AY668996
Ornithodoros_coriaceus_AY668997
Ornithodoros_coriaceus_AY668999
Ornithodoros_coriaceus_AY668975
Ornithodoros_coriaceus_AY669023
Ornithodoros_coriaceus_AY669024
Ornithodoros_coriaceus_AY668962
Ornithodoros_coriaceus_AY669000
Ornithodoros_coriaceus_AY669021
Ornithodoros_coriaceus_AY669022
Ornithodoros_coriaceus_AY669019
Ornithodoros_coriaceus_AY668994
Ornithodoros_coriaceus_AY669013
Ornithodoros_coriaceus_AY669015
Ornithodoros_coriaceus_AY669010
Ornithodoros_coriaceus_AY669014
Ornithodoros_coriaceus_AY669011
Ornithodoros_coriaceus_AY669016
Ornithodoros_coriaceus_AY669012
Ornithodoros_coriaceus_AY668995
Ornithodoros_coriaceus_AY669018
Ornithodoros_coriaceus_AY668988
Ornithodoros_coriaceus_AY668987
Ornithodoros_coriaceus_AY668991
Ornithodoros_coriaceus_AY668984
Ornithodoros_coriaceus_AY668982
Ornithodoros_coriaceus_AY669005
Ornithodoros_coriaceus_AY669002
Ornithodoros_coriaceus_AY669006
Ornithodoros_coriaceus_AY669001
Ornithodoros_coriaceus_AY668985
Ornithodoros_coriaceus_AY668986
Ornithodoros_coriaceus_AY669003
Ornithodoros_coriaceus_AY668960
Ornithodoros_coriaceus_AY668958
Ornithodoros_coriaceus_AY668989
Ornithodoros_coriaceus_AY668964
Ornithodoros_coriaceus_AY668968
Ornithodoros_coriaceus_AY668969
Ornithodoros_coriaceus_AY669020
Carios_capensis_JQ824325
Carios_capensis_JQ824324
Carios_capensis_JQ824317
Carios_capensis_JQ824310
Carios_capensis_JQ824296
Carios_capensis_JQ824307
Carios_capensis_JQ824311
Carios_capensis_JQ824298
0.3
